# Development of a T-cell activation-related module with predictive value for the prognosis and immune checkpoint blockade therapy response in glioblastoma

**DOI:** 10.7717/peerj.12547

**Published:** 2021-12-22

**Authors:** Zihao Yan, Siwen Chu, Chen Zhu, Yunhe Han, Qingyu Liang, Shuai Shen, Wen Cheng, Anhua Wu

**Affiliations:** 1 Department of Neurosurgery, The First Hospital of China Medical University, Shenyang, Liaoning, China; 2 Department of Ultrasound, The First Hospital of China Medical University, Shenyang, Liaoning, China

**Keywords:** Glioblastoma, T-cell activation, Immunotherapy, Tumor microenvironment, Immune checkpoint blockade

## Abstract

**Background:**

Despite the rise in the use of immune checkpoint blockade drugs (ICBs) in recent years, there are no ICB drugs that are currently approved or under large-scale clinical trials for glioblastoma (GBM). T-cells, which mainly mediate adaptive immunity, are an important part of the tumor immune microenvironment. The activation of T-cells in tumors plays a key role in evaluating the sensitivity of patients to immunotherapy. Therefore, we applied bioinformatics approaches to construct a T-cell activation related risk score to study the effect of the activation of T-cells on the prognosis and ICB response of patients with GBM.

**Materials and Methods:**

This study collected TCGA, CGGA, and GSE16011 glioma cohorts, as well as the IMvigor210 immunotherapy dataset, with complete mRNA expression profiles and clinical information. GraphPad Prism 8 and R 3.6.3 were used for bioinformatics analysis and plotting.

**Results:**

The activation of T-cells in patients with GBM is characterized by obvious heterogeneity. We established a T-cell activation-related risk score based on five univariate Cox regression prognostic genes (CD276, IL15, SLC11A1, TNFSF4, and TREML2) in GBM. The risk score was an independent risk factor for poor prognosis. The overall survival time of patients in the high-risk group was significantly lower than in the low-risk group. Moreover, the high-risk score was accompanied by a stronger immune response and a more complex tumor immune microenvironment. “Hot tumors” were mainly enriched in the high-risk group, and high-risk group patients highly expressed inhibitory immune checkpoints (PD1, PD-L1, TIM3 etc.). By combining the risk and priming scores we obtained the immunotherapy score, which was shown to be a good evaluation index for sensitivity to GBM immunotherapy.

**Conclusions:**

As an independent risk factor for poor prognosis, the T-cell activation-related risk score, combined with other clinical characteristics, could efficiently evaluate the survival of patients with GBM. The immunotherapy score obtained by combining the risk and priming scores could evaluate the ICB response of patients with GBM, providing treatment opportunities.

## Introduction

Glioma is the most common malignant tumor of the central nervous system (CNS). According to its histological–molecular classification, it is divided into low-grade glioma (LGG, WHO I-II grade) and high-grade glioma (HGG, WHO III-IV grade) (Wesseling & Capper, 2018; Nabors et al., 2020). Glioblastoma (GBM, WHO IV grade) is the most malignant among gliomas, and is associated with the worst prognosis (Hernández-Vega et al., 2020), due to its rapid progression, strong capacity for proliferation and invasion, and its recurrence-prone characteristics. Even in patients receiving standard radiotherapy and chemotherapy after surgery, the median survival time is only about 14 months (Thorsson et al., 2018). Of note, GBM tumors are characterized by the accumulation of a large number of immune cells due to the secretion of chemokines (Broekman et al., 2018) by the tumor and the occurring angiogenesis during tumorigenesis (Hardee & Zagzag, 2012), as well as due to the tumor progression-induced destruction of the blood-brain barrier (Schulz et al., 2019; Arvanitis, Ferraro & Jain, 2020). Most of these infiltrating immune cells have lost their tumor-killing ability (Wherry & Kurachi, 2015; Yost et al., 2019), and are characterized by the high expression of inhibitory immune checkpoints (Sakuishi et al., 2010; Rooney et al., 2015).

A significant heterogeneity has been reported in the tumor immune microenvironment between different grades of gliomas as well as between patients with the same grade (Zhang et al., 2017). Therefore, research on tumor microenvironments appears to be the key for individualized treatment (Jia et al., 2018). Although the main immune cells found in the brain are macrophages and microglia, increasing studies (Rooney et al., 2015; Zhai et al., 2020) have shown that T-cells are closely related to the tumorigenesis of GBMs. T-cells are known to mainly mediate adaptive immune responses. Different subtypes of T-cells have been shown to exert different roles in GBMs. For example, T-regs and Th2 cells were considered to inhibit antitumor immunity (Mahata et al., 2014; Jia et al., 2018), whereas CD8+ and CD4+ T-cells were reported to act as cytotoxic antitumor immune cells (Charoentong et al., 2017). In fact, as a more complex tumor microenvironment indicates a worse prognosis, the tumor-killing ability of many such T-cells might have been suppressed in GBM (Chen & Flies, 2013; Im et al., 2016).

In recent years, there has been an increasing number of studies using immune checkpoint blockade (ICB) factors, such as programmed cell death 1 receptor (PD1), programmed cell death ligand 1 (PD-L1), and cytotoxic T-lymphocyte associated protein 4 (CTLA4) as therapeutic targets (Bodor, Boumber & Borghaei, 2020), with significant effects being achieved in diseases such as melanoma, lung, breast, and bladder cancers (Le et al., 2017; Cyriac & Gandhi, 2018). However, because of the unclear immunotherapy effect in GBM, and its cytotoxicity to normal brain tissue, most studies were only limited to basic experimental research (Lim et al., 2018). Studies on immunotherapy of patients with GBM have been limited; therefore, large-scale clinical data on ICB-targeting therapies cannot be obtained. With the development and application of Next-generation sequencing (NGS) technology (Slatko, Gardner & Ausubel, 2018), large-scale sampling databases have been used to explore tumor microenvironments. The application of RNA sequencing (Stark, Grzelak & Hadfield, 2019) has allowed us to predict the sensitivity of patients to immunotherapies, and has also provided possibilities to develop more rapid and individualized treatment strategies for patients with GBM (Jiang et al., 2018; Fu et al., 2020).

The activation of T-cells has been shown to be closely related to the tumor microenvironment, immunotherapy, and prognosis of tumors (Sade-Feldman et al., 2018). Many studies are currently investigating the mechanism of activating the tumor-killing effect of T-cells (Johnson et al., 2015; Dangaj et al., 2019) and the reasons behind the immunosuppression of T-cells in tumors (Pauken et al., 2016; Yost et al., 2019). However, the overall relationship between the activation of T-cells and prognosis, as well as between the tumor microenvironment and the sensitivity to immunotherapy remains unclear in GBM.

In this study, we developed a T-cell activation-related risk score in glioblastomas through large-scale bioinformatics analyses, which is an independent poor prognostic factor. We also profiled the relationship between the risk score and the tumor microenvironment in patients with GBM. Furthermore, an immunotherapy score was constructed to predict the ICB response of patients with GBM. We identified that patients with high immunotherapy scores may be more sensitive to immunotherapy, with certain clinical translation value.

## Materials and Methods

### Patient data

A total of 672 patients with GBM and 1,151 patients with LGG were included in this study. Data on all patients were obtained from the following four databases: TCGA RNA-seq cohort (https://tcga-data.nci.nih.gov, Version 19.0), CGGA325 RNA-seq cohort (http://www.cgga.org.cn, updated on Nov 28, 2019) (Zhao et al., 2021), CGGA693 RNA-seq cohort (http://www.cgga.org.cn, updated on Nov 28, 2019) (Zhao et al., 2021), and GSE16011 microarray (updated on Mar 14, 2014). The TCGA database was used as the discovery cohort here, the CGGA325 cohort was used as the internal validation set, and CGGA693 and GSE16011 were used as the external validation sets. Samples in all datasets contained complete clinical information, such as survival, age, grade, and IDH1 mutational status. The immunotherapy dataset used was IMvigor 210 (IMvigor210CoreBiologies package), which contained 298 bladder cancer samples. The dataset included gene expression profiles, immunotherapy data, and overall survival. Overall survival (OS) was calculated from the date of diagnosis to the date of death or final follow-up. All RNA-seq expression profiles were in FPKM format, and the microarray profile was calculated by the average value of probes for each gene.

### Establishment and standardization of T-cell activation-related risk score and T-cell activation priming score

The genes in the tumor-related T-cell activation gene set were subjected to univariate Cox regression analysis to obtain genes with prognostic value (Cox-*P* < 0.05) in the GBM cohorts (Table S1). From the intersection of TCGA GBM and CGGA325 GBM Cox results, we obtained five genes: CD276, interleukin-15 (IL-15), solute carrier family 11 member 1 (SLC11A1), TNF superfamily member 4 (TNFSF4), and triggering receptor expressed on myeloid cells like 2 (TREML2). Subsequently, the risk score was established based on the hazard ratio (HR) of these genes:

${\rm {Risk\; Score = [LN(HR)  expression]}}$

We used the R GSVA package to get a single sample gene set enrichment analysis (ssGSEA) score of the gene set GO:0002291 (T-cell activation *via* T-cell receptor contact with antigen bound to MHC molecule on antigen presenting cell, http://www.broadinstitute.org/gsea/index.jsp) for each sample (Hänzelmann, Castelo & Guinney, 2013). We then obtained the T-cell activation priming score.

The min-max normalization method was used to standardize the two scores so that both would be distributed in the (0,1) interval. The specific formula was as follows:

${\rm {x^* = (x-min)/(max-min)}}$

x* is the standardized score, x is the original score, min is the minimum value in the dataset, and max is the maximum value in the dataset.

### Establishment of immunotherapy score

We used the standardized priming score to subtract the standardized risk score. Consecutively, a Z-score standardization was performed to obtain a standard normal distribution score, which we termed immunotherapy score. The specific formula was as follows:

${\rm {Immunotherapy\; Score = Z-score\ (Priming\; Score - Risk\; Score)}}$

${\rm {Z-score = (x-)/}}$

x is the original value, μ is the mean of the sample, and δ is the standard deviation of the sample.

### Survival analysis

We used the R survival package and GraphPad Prism 8 to perform univariate and multivariate Cox regression analyses of patients, as well as to analyze the differences in survival between the high- and low-risk groups and the high- and low-immunotherapy score groups. We used the R survival ROC package to draw a nomogram, and then applied calibration plots to validate it.

### Gene ontology analyses, kyoto encyclopedia of genes and genomes pathway analyses, and gene set enrichment analyses

We used the R limma package to calculate the differential expression of genes between high- and low-risk groups. Genes with an FDR < 0.05 were adopted and intersections were taken in the four datasets. We also used the R clusterProfiler, org.Hs.eg.db, enrichplot, and ggplot2 packages to perform GO and KEGG pathway enrichment analyses and to plot the results. We then performed GSEA analyses between the high- and low-risk groups on immune-related gene sets. Finally, we applied the R GSVA package to analyze 22 tumor-related gene-programs using the ssGSEA function.

### Analyses of tumor purity and tumor immune microenvironment

The stromal score and the immune score were calculated using the R Estimate package, while the tumor purity was calculated according to the method previously described (Zhang et al., 2017). The relative quantitation of multiple immune cells in GBM was achieved in R 3.6.3 using the Metagenes method. Accordingly, immune cells were divided into three types: antitumor effect type, tumor-promoting immunosuppression type, and intermediate type (Charoentong et al., 2017). An antitumor immunity score and a protumor suppression score based on various types of immune cells were calculated for each sample. Differences in the results of the MCP counter were used to validate the above findings (Becht et al., 2016).

### Tumor microenvironment immune types and hot and cold tumors

According to the level of Cytolytic activity (CYT) and PD-L1, patients were divided into four immune subtypes: TMIT I to IV (Rooney et al., 2015). In particular, TMIT I and IV subtypes were termed hot tumor, whereas TMIT II and III subtypes were termed cold tumor (Teng et al., 2015; Ock et al., 2016; Chen et al., 2017).

### Statistical analysis

We mainly used R 3.6.3 and GraphPad Prism 8 for statistical analysis and plotting of the data. Univariate and multivariate Cox regression analysis were performed for survival analysis. According to the median risk score, patients were divided into high- and low-risk groups. A two-sample *t*-test was used to determine whether there was a significant difference between two groups. The Kaplan–Meier survival analysis and log-rank test were used to evaluate the survival difference between two groups. Calibration plots and the C-index were used to evaluate the accuracy of the nomogram model. FDR was used to evaluate the significance of GSEA results. Pearson linear correlation was used to calculate correlations, where r was the correlation coefficient. Chi-square test was used to compare the differences in immune subtypes and the sensitivity to ICB therapy between two groups (Wang et al., 2021a). In the above statistical analyses, *P* < 0.05 and FDR < 0.25 were regarded as statistically significant.

## Results

### Establishment of T-cell activation-related risk score and clinical characteristics

As is known, the activation of T-cells in tumors has been closely related to the sensitivity of patients to immunotherapy (Kim, Herbst & Chen, 2018), and hence has been found to be heterogeneous in different samples. In order to further explore the differences in the activation of T-cells in gliomas, we obtained a total of 15 gene sets, including a T-cell activation gene set and its derivative terms from the GSEA website (http://www.gsea-msigdb.org/gsea) to be used for ssGSEA analysis (Zhu et al., 2019). Our results showed that the ssGSEA enrichment score of the T-cell activation gene set was generally higher in GBM relative to that in LGG. In addition, we found that the score was heterogeneous in the same grade gliomas (Fig. S1A).

**Figure 1:**
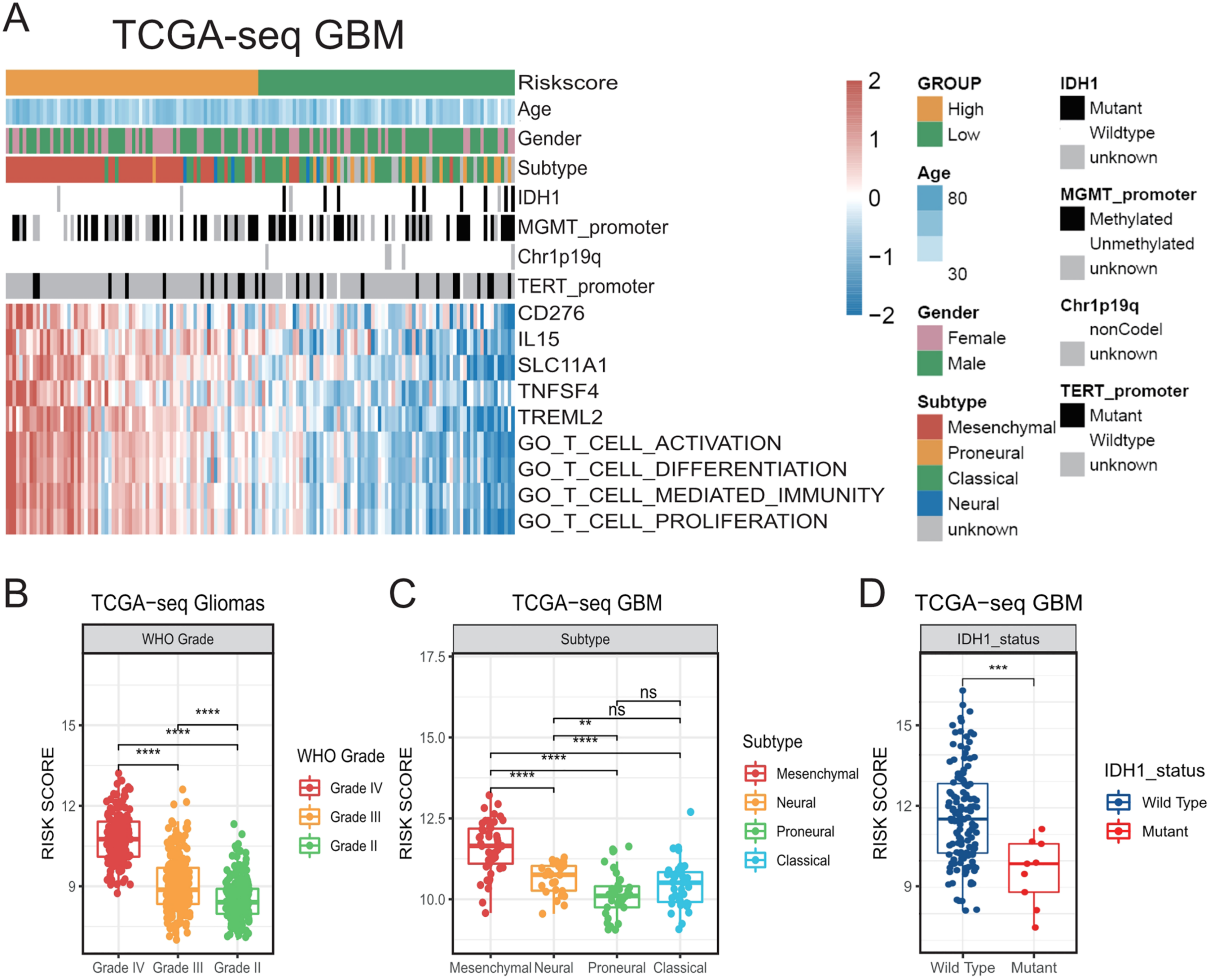
The establishment of T cell activation related risk score and relationships between the risk score and clinical characteristics in TCGA cohort. (A) The heatmap of risk score’s five genes expression and other clinical characteristics distribution of GBM patients. (B) High risk score was distributed in higher grade gliomas (Student’s t test, ** means *P* < 0.01, *** means *P* < 0.001, **** means *P* < 0.0001). (C–D) The higher risk score patients were mainly concentrated in the mesenchymal subtype (C) and IDH1 wild type (D) in GBM (Student’s t test, ** means *P* < 0.01, *** means *P* < 0.001, **** means *P* < 0.0001).

In order to further investigate the relationship between the activation of T-cells and the clinical features in GBM, we screened out genes in GSEA T-cell activation gene set that were confirmed to be tumor-related through literature search in PubMed. And then, we obtained a tumor-related T-cell activation gene set containing 262 genes, which is summarized in Table S1. We subjected the genes to univariate Cox regression analysis, and from the intersection of TCGA GBM and CGGA325 GBM Cox results we obtained five stable prognostic genes (Cox-*P* < 0.05): CD276, interleukin-15 (IL-15), solute carrier family 11 member 1 (SLC11A1), TNF superfamily member 4 (TNFSF4), and triggering receptor expressed on myeloid cells like 2 (TREML2). We found that all these genes were risk indicators for poor prognosis with HR > 1, and were positively correlated to the ssGSEA enrichment score of T-cell activation-related gene sets on the GSEA database (Fig. 1A). Therefore, we constructed a prognostic risk model related to the activation of T-cells based on the sum of the LN(HR)* expression of these five genes, as follows: Risk Score = (0.5258 × CD276 expression) + (0.1477 × IL-15 expression) + (0.2178 × SLC11A1 expression) + (0.2346 × TNFSF4 expression) + (0.2192 × TREML2 expression) (Cheng et al., 2016; Zhu et al., 2019).

We observed that our generated risk score showed a significant positive correlation with the ssGSEA enrichment score (all *P* < 0.001, Figs. S1B–S1I), indicating that it could be a good indicator of the relative status of the activation of T-cells in GBM cohorts.

Then, we compared the risk scores on different WHO grades of gliomas in TCGA. We accordingly found that the risk score of GBMs was higher than that of LGGs (Fig. 1B). We also found that the risk score was prominently higher in the mesenchymal subtype compared with that in other subtypes in TCGA GBM (Figs. 1A, 1C). In addition, higher-risk patients were demonstrated to be mainly characterized by the presence of the IDH1 wild-type (Figs. 1A, 1D) (Venteicher et al., 2017). We subsequently confirmed all the above results in validation cohorts (Fig. S2). Our results suggest that the risk score was related to a number of clinical characteristics with poor prognosis, and a higher risk score might indicate a more complex tumor microenvironment.

### Significant prognostic value of risk score

**Figure 2:**
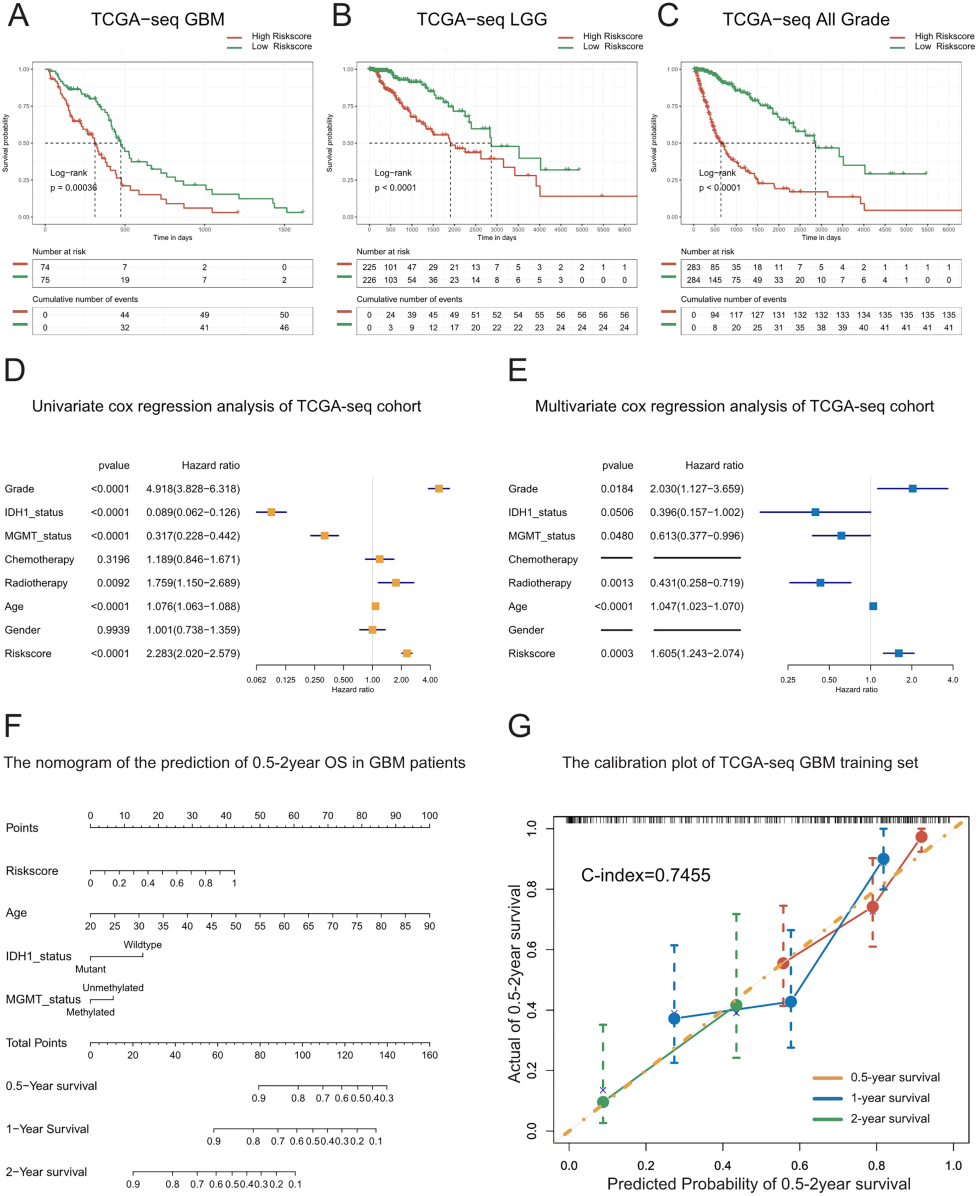
Prognostic value of the T cell activation related risk score in TCGA cohort. (A–C) Patients with high risk score in GBM(A), LGG(B) and All grades (C) had poor prognosis. ((A) *P*-value = 0.0004, (B) *P*-value < 0.0001, (C) *P*-value < 0.0001, Log-rank test). (D–E) Forest plots of univariate(D) and multivariate (E) Cox regression analysis of the risk score. (F) The nomogram for the prediction of overall survival probability of GBM patients. (G) The calibration plot of TCGA-seq GBM training set.

To further evaluate the prognostic value of the T-cell activation-related risk score, we divided the patients into two groups according to the median value of the risk score, and compared the differences in survival between the two groups. We used TCGA as the discovery cohort, CGGA325 as the internal validation cohort, and CGGA693 and GSE16011 as the external validation cohorts. We found that the overall survival time of patients with GBM in the high-risk group was significantly lower than that in the low-risk group. This finding was further confirmed in the validation cohorts (Fig. 2A, Figs. S3A–S3C). Moreover, we noticed that the risk score exhibited also a remarkable prognostic value in LGG and all-grade glioma (Figs. 2B–2C, Figs. S3D–S3I).

To explore whether the risk score is a continuous independent prognostic indicator, we performed univariate and multivariate Cox regression analyses. We found that the risk score was a robust indicator of poor prognosis, independent of other clinical characteristics such as grade, IDH1 mutational status, MGMT promoter methylation, radiotherapy, and age (HR = 1.605, 95% CI [1.243−2.074], *P* = 0.0003). We further confirmed these results in the validation sets (Figs. 2D–2E, Figs. S4A–S4F).

In order to strengthen the survival prediction and clinical translation of our risk score, we standardized the risk scores of TCGA patients with GBM using the min-max normalization method, and established a nomogram to predict the 0.5-2-year survival probability of patients with GBM (Fig. 2F). We observed that the C-index of the nomogram was 0.7455. In addition, the calibration plot showed that the predicted survival probability was highly consistent with the actual survival rate. The nomogram also showed high survival prediction accuracy in the validation sets (Fig. 2G, Figs. S4G–S4H). These results indicate that the risk score had high survival prediction accuracy and considerable clinical translation value.

### High-risk score indicated a stronger immune response

**Figure 3:**
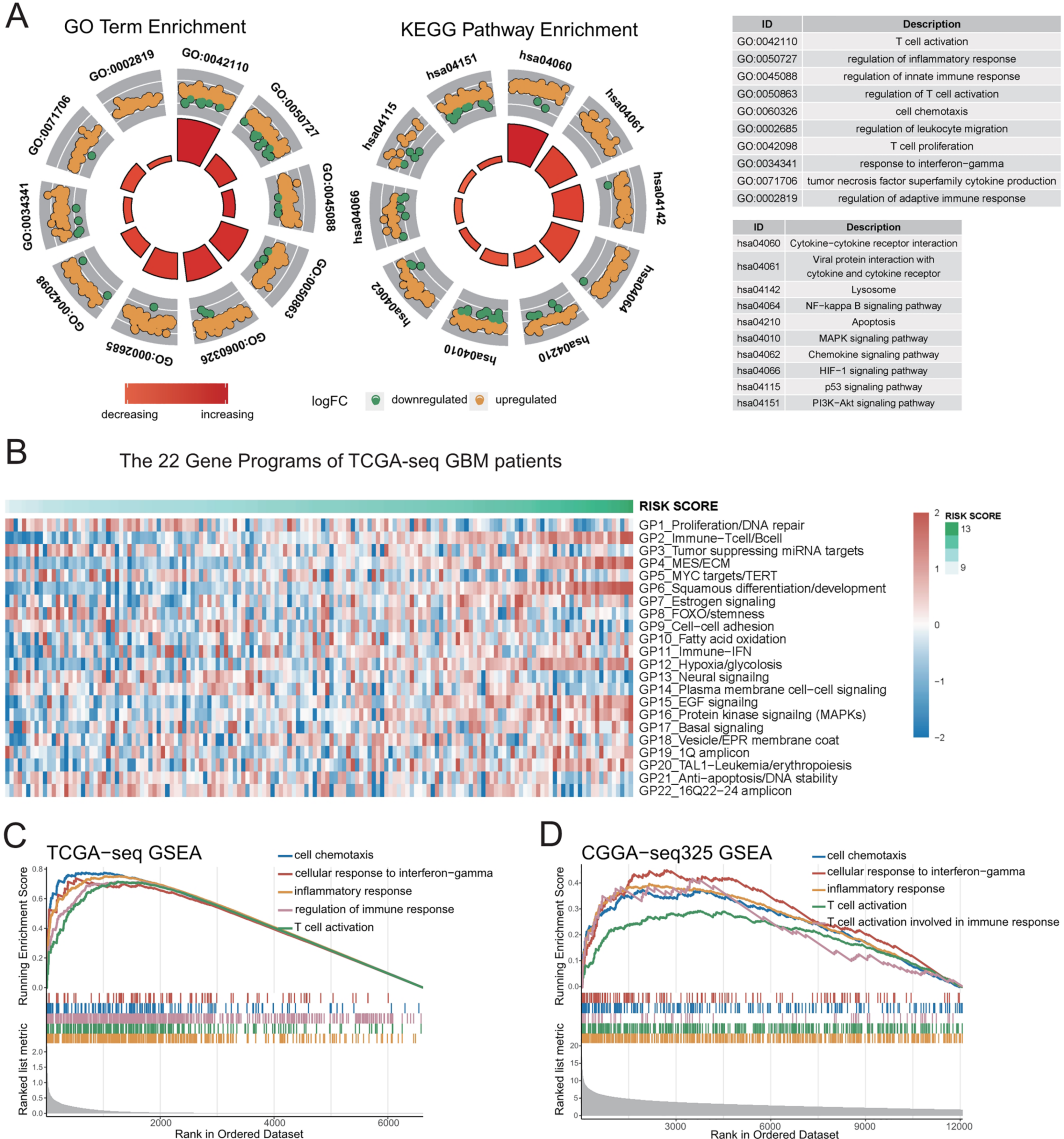
Genetic functional enrichment analysis of the differentially expressed genes between the high and low risk score groups. (A) GO and KEGG pathway enrichment analyses of the differentially expressed genes between the high and low risk score groups. (B) The ssGSEA score of 22 Gene Programs of TCGA-seq GBM patients. (C–D) TCGA GBM(C) and CGGA-325 GBM(D) GSEA analyses showed that immune-related gene sets were significantly enriched in the high risk score group.

To study the major differences in biological processes between the high- and low-risk groups, we obtained the differentially-expressed genes (FDR < 0.05) between the high- and low-risk groups using the limma package, and took the intersection of genes in the four datasets for enrichment analyses. The results of GO and KEGG pathway analyses reveal that immune-related biological processes (Fig. 3A, Figs. S5A–S5B, Table S2), such as inflammation, immune response regulation, interferon gamma, cell chemotaxis, lymphocyte migration, as well as some typical carcinogenic pathways, such as NF-kappa B, PI3K-AKT, P53, and apoptosis pathways were the mainly enriched processes in the high-risk group.

To further demonstrate the correlation of the risk score to certain biological processes, we performed ssGSEA on 22 canonical to cancer gene-programs (GPs). Our results showed that five gene-programs, the GP2_Immune-Tcell/Bcell, GP4_MES/ECM, GP11_Immune-IFN, GP12_Hypoxia/glycolosis, and GP15_EGF Signaling were related to the risk score in both the discovery and validation cohorts. Consistent with the above results, we found that all five gene-programs were correlated with the immune response (Fig. 3B, Figs. S5C–S5F).

Concomitantly, we performed gene set enrichment analysis (GSEA) on five immune-related terms, and found that these immune-related terms were enriched in the high-risk group (Figs. 3C–3D, Figs. S5G–S5H, Table S2). These results indicated that the high-risk group might have a more complex immune response. This finding verifies the potential differences in tumor immune microenvironments between the high- and low-risk groups.

### High-risk score indicated a more complex and immune escaped tumor microenvironment

**Figure 4:**
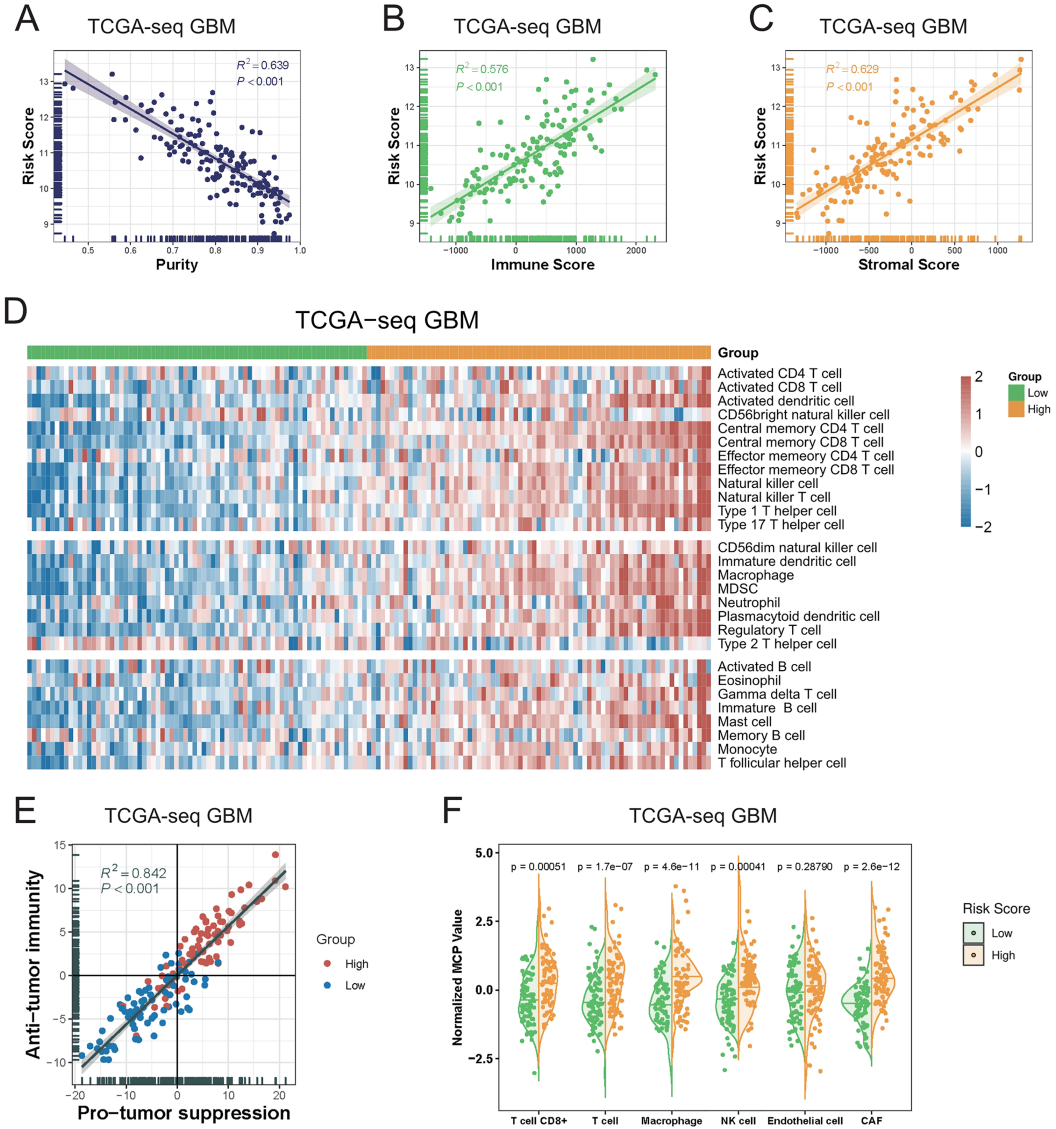
The relationship between T cell activation related risk score and tumor microenvironment in TCGA-seq GBM cohort. (A–C) The risk score was negatively correlated with tumor Purity ((A), Spearman correlation, R^2^ = 0.639, *P* < 0.001), and positively correlated with Immune Score ((B), Spearman correlation, R^2^ = 0.576, *P* < 0.001) and Stromal Score ((C), Spearman correlation, R^2^ = 0.629, *P* < 0.001). (D) The heatmap of Metagenes results showed that high risk score was associated with increasing multiple immune cells in the tumor. (E) Metagenes results showed that there was a positive correlation between the anti-tumor immunity score and the pro-tumor suppression score, and both were higher in the high risk score group (Spearman correlation, R^2^ = 0.842, *P* < 0.001). (F) MCP counter compared differences in various types of immune cells between the high and low risk score groups (Student’s t test).

As the immune-related gene sets were demonstrated to be the mainly enriched differentially-expressed genes in the high-risk group, we investigated the relationship between the T-cell activation-related risk score and the tumor microenvironment. First, we found that the risk score showed a strongly positive correlation with tumor purity, whereas negative correlations with the immune and stromal scores. We also obtained the similar result in the validation sets (Figs. 4A–4C, Fig. S6). This finding indicated that a higher risk score was associated with lower tumor purity and a more complex tumor microenvironment.

We next explored the relationship between the risk score and various immune cellular components in the tumor microenvironment. Our Metagenes results showed that the levels of various immune cells in the tumor were increased in parallel with an increase in the risk score. According to their effects in tumors, immune cells were divided into three types: antitumor immunity type, protumor suppression type, and intermediate type. We observed that as the risk score increased, the levels of antitumor immunity and protumor suppression cells in tumors were concomitantly increased (Fig. 4D, Fig. S7) (Li et al., 2018).

We detected a significant positive correlation between the antitumor immunity and protumor suppression scores in GBM, as calculated using the Metagenes method. In particular, we found that both immune effects were enhanced with an increase in the risk score (Fig. 4E, Fig. S8). We then used the MCP counter, a tumor immune microenvironment component analysis tool, to validate our results, and found that the immune cell content was increased in tumors in parallel with an increase in the risk score (Fig. 4F, Fig. S9).

### Hot tumors were mainly concentrated in the high-risk group

**Figure 5:**
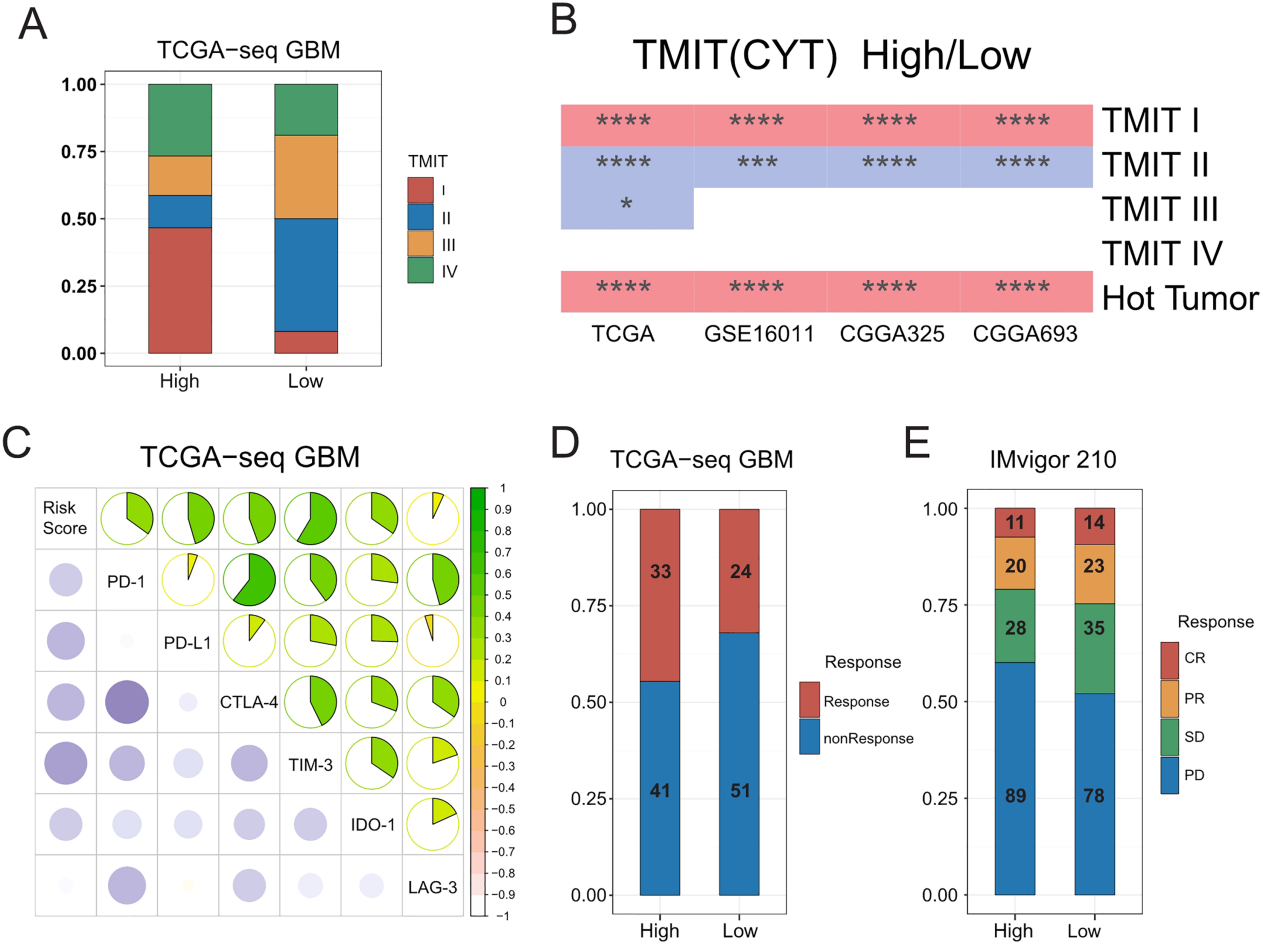
Relationships between the risk score and the hot and the cold tumors, immune checkpoints expression, and the sensitivity of patients to immunotherapy. (A) Proportion differences of Tumor Microenvironment Immune Types between the high and low risk score groups in TCGA GBM patients. (B) Proportion differences of Tumor Microenvironment Immune Types and the hot and the cold tumors between the high and low risk score groups’ patients in TCGA, GSE16011, CGGA325 and CGGA693 cohorts (red color: the high-risk group had a higher proportion than the low-risk group. Blue color: the low-risk group had a higher proportion than the high-risk group. * means *P* < 0.05, *** means *P* < 0.001, **** means *P* < 0.0001). (C) The risk score showed a positive correlation with the expression level of immune checkpoints: PD-1, PD-L1, CTLA-4, TIM-3, IDO-1 and LAG-3 in TCGA GBM cohort. (D) The risk score was not related to TIDE results in TCGA GBM cohort (*P*-value = 0.1577, chi-square test). (E) The sensitivity to immunotherapy was not associated with T cell activation related risk score in IMvigor 210 immunotherapy dataset (CR, PR, SD were considered as Response, PD was considered as nonResponse. *P*-value = 0.1943, chi-square test).

A previous pan cancer analysis divided tumors into four tumor microenvironment immune types based on the expression of PD-L1 and level of Cytolytic activity (CYT). Accordingly, type I and type IV with high levels of CYT were considered hot tumors, whereas type II and type III with low levels of CYT were considered cold tumors. Based on this, we initially classified GBMs according to the expression of PD-L1 and level of CYT. We found that the proportion of type I patients in the high-risk group was significantly higher than that in the low-risk group, whereas type II patients mainly in the low-risk group. We further observed that hot tumors were mainly concentrated in the high-risk group, whereas cold tumors were mainly concentrated in the low-risk group. Finally, we confirmed all these results in validation sets (Figs. 5A–5B, Fig. S10A).

### Significant correlations between the risk score and expression of immune checkpoints

A number of recent studies (Tumeh et al., 2014; Du et al., 2020; Noman et al., 2020) showed that compared with cold tumors, hot tumors have increased sensitivity to immunotherapy, thus allowing for more immunotherapy opportunities. In addition, the higher expression of immune checkpoints such as PD-1 and PD-L1 has indicated that hot tumors are more suitable for targeted immunotherapy (Wang et al., 2016). We therefore calculated the correlation between the risk score and the expressions of some classic immune checkpoints, and found that the risk score was positively correlated with immune checkpoints, such as PD-1, PD-L1, CTLA-4, hepatitis A virus cellular receptor 2 (TIM3), and indoleamine 2, 3-dioxygenase 1 (IDO1) (Fig. 5C, Figs. S10B–S10D).

We inferred that the T-cell activation-related risk score might be used to evaluate the ICB response of patients with GBM (Mi et al., 2018; Anghileri et al., 2021). To this end, we used the TIDE mean (http://tide.dfci.harvard.edu/) to predict the ICB response of patients with GBM, and found that there was no difference in the proportion of patients that were sensitive to immunotherapy between the high- and low-risk groups (Fig. 5D). No FDA-approved ICB drugs for GBM are currently available. Furthermore, as no public large-scale immunotherapy clinical trial has ever been performed, we used IMvigor 210, the most widely used public immunotherapy dataset, to validate our TIDE results. Not surprisingly, we found that the T-cell activation-related risk score could not distinguish between the sensitivity of patients to immunotherapy among the two groups (Fig. 5E). Although a higher risk score was associated with an increased infiltration of immune cells in tumors, we noticed that the risk score was also significantly positively correlated with the expression of TIM-3 and IDO1 (Fig. 5C, Figs. S10B–S10D) (Wherry & Kurachi, 2015; Li et al., 2017). This finding suggests the exhaustion of most activated T-cells in tumors due to continuous stimulation by antigens (Satpathy et al., 2019). Recent studies have shown that immune checkpoint blockade therapy cannot restore the tumor-killing ability of exhausted T-cells (Yost et al., 2019). Therefore, combined with the cancer-immunity cycle (Chen & Mellman, 2013), the content of priming but not exhausted T-cells in tumors might be the key to evaluating the sensitivity of GBMs to immunotherapy.

### Combining the priming and risk scores to predict the ICB response of GBMs.

**Figure 6:**
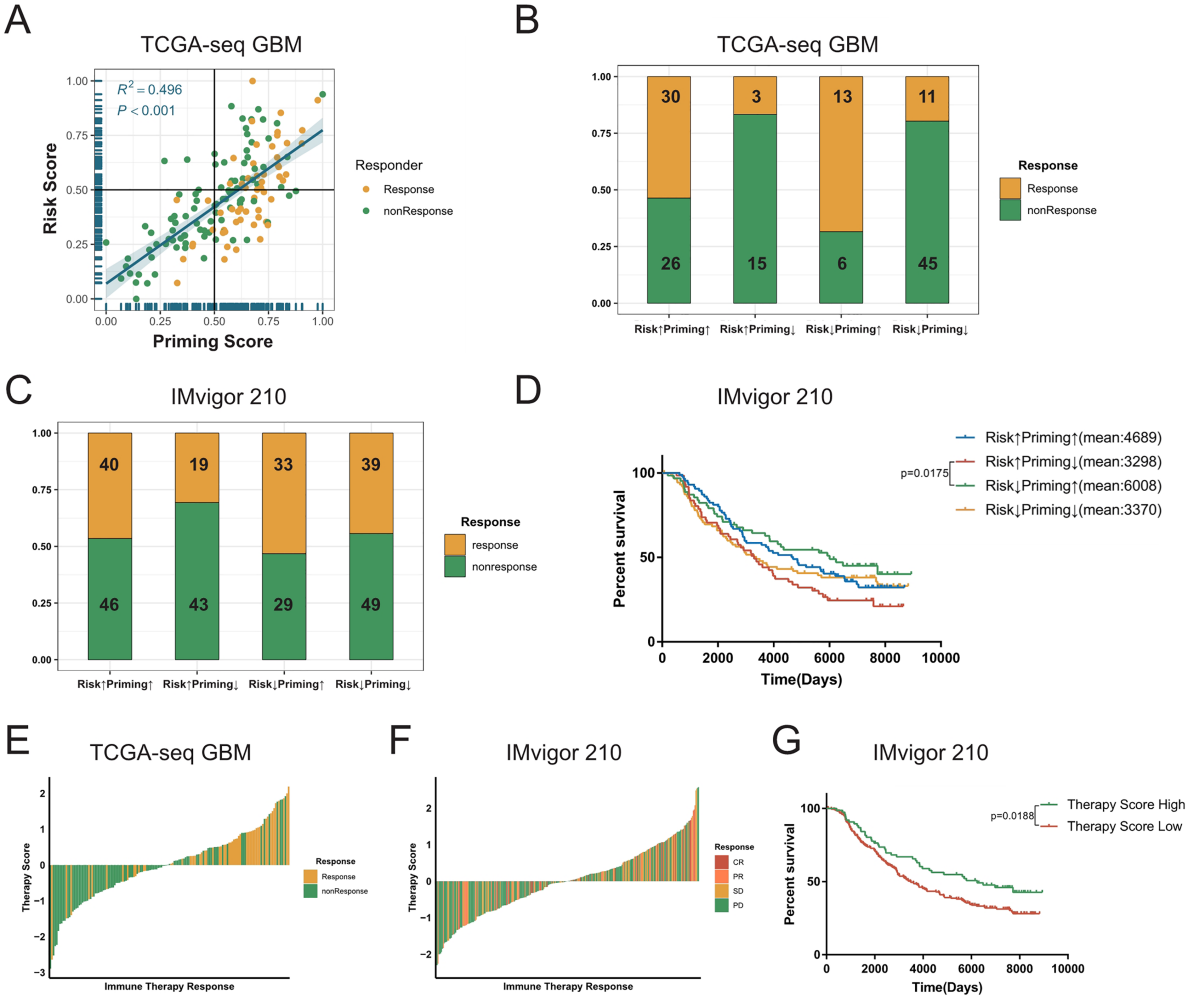
Combined the T cell activation priming score and the risk score to predict the sensitivity of patients with GBM to immunotherapy. (A) The priming score showed a positive correlation with the risk score in TCGA GBM cohort, and TIDE results showed that patients were sensitive to immunotherapy while the priming scores were high and the risk scores were low (Spearman correlation, R^2^ = 0.496, *P* < 0.001). (B) There was a significant difference in TIDE results between the high-risk/low-priming group and the high-priming/low-risk group in TCGA GBM (*P*-value = 0.0045, chi-square test). (C) There was also a significant difference in the sensitivity to immunotherapy between the high-risk/low-priming group and the high-priming/low-risk group in IMvigor 210 immunotherapy dataset (*P*-value = 0.0180, chi-square test). (D) Compared to high-risk/low-priming patients, there was a longer survival of high-priming/low-risk patients in IMvigor 210 immunotherapy dataset (Risk^↓^Priming^↑^mean = 6,008 days; Risk^↑^Priming^↓^mean = 3,298 days. *P*-value = 0.0175, Log-rank test). (E) The immunotherapy score was significantly associated with TIDE results in TCGA GBM cohort (*P*-value < 0.0001, chi-square test). (F) The immunotherapy score was significantly associated with the sensitivity to immunotherapy in IMvigor 210 immunotherapy dataset (*P*-value = 0.0276, chi-square test). (G) In IMvigor 210 dataset, there was a significantly longer overall survival in the high-therapy score group compared with those patients in the low-therapy score group (*P*-value = 0.0188, Log-rank test).

The process of TCR binding to the antigen-MHC molecular complex on antigen presenting cells (APCs) is the initiation signal of the activation of T-cells (Chowell et al., 2018; Gu et al., 2021). We obtained gene signature data on the activation of T-cells *via* contact of their T-cell receptor with antigen bound to MHC molecules on antigen presenting cells (GO:0002291) from the GSEA database, and performed ssGSEA on GBM samples to get the priming score. Because the activation of T-cells in GBMs was significantly positively correlated with exhaustion markers, we assumed that a higher risk score in patients would be associated with greater immunosuppression in tumors (Fig. 5C, Figs. S8D–S8F). Therefore, we combined two scores to evaluate whether there were any differences in the sensitivity to ICBs among patients with GBM (Stathias et al., 2018; Wang et al., 2021b). First, we detected a positive correlation between the priming and risk scores in the discovery cohort (R^2^ = 0.496, *P* < 0.001, Fig. 6A). Our TIDE prediction results revealed that the low-risk/high-priming group had the highest proportion of ICB-sensitive patients, whereas the high-risk/low-priming group had the lowest proportion of ICB-sensitive patients (*P* = 0.0045, chi-square test, Fig. 6B). We obtained the same results in the validation cohorts and the IMvigor 210 immunotherapy dataset (Fig. 6C, Figs. S11A–S11F). Hence, patients in the low-risk/high-priming group were demonstrated to significantly benefit from ICBs. We also observed that compared with the high-risk/low-priming patients, low-risk/high-priming patients had a longer survival in the IMvigor 210 immunotherapy cohort (Fig. 6D).

To further explore the tumor-killing potential of activated T-cells in GBMs, we standardized the priming and risk scores and then subtracted them. After further standardizing by Z-Score, we got immunotherapy score. According to the median immunotherapy score, patients were divided into a high- and low-responding group. We found that in the TCGA and validation cohorts, the TIDE predictive ICB response rate of patients with GBM with high therapy scores was significantly higher than that of patients with low therapy scores (*P* < 0.0001, chi-square test, Fig. 6E). Importantly, this result was also verified in validation sets (Figs. S11G–S11I). Similarly, we noticed that in the IMvigor 210 cohort, the ICB response rate in the high-immunotherapy group was significantly higher than that in the low-therapy group (*P* = 0.0276, chi-square test, Fig. 6F). Patients with high-therapy score had better prognostic value with longer OS (*P* = 0.0188, Log-rank test, Fig. 6G). Therefore, the immunotherapy score could be a good indicator of the sensitivity to ICB therapy, and patients with high immunotherapy scores would be more likely to benefit from ICBs.

## Discussion

Increasing number of studies have shown that the central nervous system is not an immune-privileged organ, but rather is characterized by a large number of innate and adaptive immune responses. Tumor cells, stromal cells, immune cells, and extracellular matrix together constitute a complex tumor immune microenvironment in GBM (Galon et al., 2006). Among them, T-cells, an important type of immune cells in GBMs that mainly participate in adaptive immune responses, play an important role in tumorigenesis and progression of tumors. Hence, T-cells appear to be the key to GBM immunotherapy. A visible heterogeneity has been reported in the activation of T-cells between different grades of gliomas, as well as between samples of the same grade. Therefore, we screened five T-cell activation-related genes with prognostic value in GBMs and constructed a Cox regression risk score based on these five genes. Our results revealed significant differences in the risk score among gliomas of different grades, different subtypes, and different molecular characteristics. In particular, the more complex tumor immune microenvironment in patients with GBM was shown to often predict a worse prognosis. Our study found that the higher risk score was associated with stronger immune responses, a more complex tumor immune microenvironment, and a worse prognosis in patients with GBM. We specifically observed that in samples with high-risk scores both the antitumor immune and protumor suppression effects were significantly enhanced. Some studies have shown that although the risk score is positively correlated with certain immunotherapy targets and enriched in hot tumors, it has also been correlated with T-cell exhaustion markers, such as TIM3 and IDO1, indicating that the stronger the activation of T-cells, the greater the exhaustion of T-cells in GBMs. Recent studies have also shown that exhausted T-cells cannot restore their tumor-killing ability (Chen & Mellman, 2013); therefore, the risk score could not potentially distinguish the TIDE-predicted ICB response. To this end, we combined the priming and risk scores to establish an immunotherapy score that would provide the potential tumor-killing ability of T-cells in patients with GBM. We found that the immunotherapy scores could distinguish between the TIDE-predicted results in all cohorts; our findings were further validated in the IMvigor 210 immunotherapy dataset. Patients with high immunotherapy scores were demonstrated to have obviously benefited from ICBs. Hence, establishment of the risk and immunotherapy scores would facilitate the effective prediction of prognosis and ICB response of patients with GBM.

We used univariate Cox regression analyses to establish a T-cell activation-related risk score in GBM, which was not been previously reported. The generated risk score could provide a satisfactory evaluation of the prognosis and tumor immune microenvironment of patients with GBM. The nomogram could precisely predict the survival time of patients, and as it has been well-validated in other cohorts, it could probably be effectively used for clinical translation. At present, the efficacy of GBM immunotherapy remains unclear, and no clinical trial on the potential benefit of patients with GBM from ICBs has ever been performed. Our immunotherapy score can efficiently evaluate the T-cell tumor-killing potential in patients with GBM and distinguish between the TIDE-predicted results, as verified in validation cohorts and the IMvigor 210 immunotherapy dataset. In addition, the immunotherapy score has a certain predictive value for the ICB therapy of patients with GBM, and could be used to provide basic rationales for GBM treatments with the possibility of further clinical applications in the future.

In this study, the risk score was significantly positively correlated with the T-cell activation GSEA gene set enrichment score, indicating that the risk score could effectively depict the relative status of the activation of T-cells in GBM samples. Even though there have been a few previous studies on the prediction of the ICB response of patients with GBM, our immunotherapy score could predict the response of patients to ICBs in multiple independent cohorts. Interestingly, the risk and therapy scores developed here, used only five and 13 genes, respectively, to evaluate the prognosis and ICB response of patients with GBM. Compared with other gene sets employing hundreds of genes, the number of genes utilized in both signatures was greatly reduced. We believe that both scores could potentially have extremely high clinical applications, facilitating the development of novel GBM treatments. However, this study has its limitations. First, this was a retrospective study, and no prospective studies were performed for validation. Second, no public GBM patient immunotherapy datasets currently exist to validate the TIDE results. Finally, we should combine single-cell sequencing to further study the interactions between activated T-cells and tumor cells in the future (Kieffer et al., 2020). Meanwhile, the mechanism underlying the activation of T-cells in GBMs by the action of these five genes should be thoroughly investigated to facilitate the improved integration of the risk score with clinical practice.

## Conclusions

In this study, we screened out five T-cells activation related genes with survival value in GBM. We used these genes to construct a T-cells activation related risk score and found it was significantly related to clinical characteristics and molecular subtypes of GBM. The higher risk score indicated worse prognosis for patients with GBM. The nomogram which we had constructed could predict the survival time of patients with high accuracy. Differentially-expressed genes between the high and low risk group were mainly enriched in immune-related pathways. A higher risk score was associated with lower tumor purity and a more complex tumor microenvironment. The high-risk group mainly had “hot tumors” and highly expressed inhibited immune checkpoints (PD1, PD-L1, TIM3 etc.). However, the risk score could not distinguish the sensitivity of patients to immunotherapy. Therefore, we conducted a joint analysis of the priming score and risk score and then obtained the therapy score. The therapy score could predict the sensitivity of patients to immunotherapy well, which provided improvement in clinical translation and therapy.

## Supplemental Information

10.7717/peerj.12547/supp-1 Supplemental Information 1 The difference in T cell activation between GBM and LGG, and correlations between T cell activation related risk score and GO_T_CELL_ACTIVATION ssGSEA enrichment score. A: GO_T_CELL_ACTIVATION and its child terms’ ssGSEA enrichment scores distributed differently in TCGA glioma patients, and were significantly higher in GBM patients. B-I: T cell activation related risk score was significantly positively correlated with GO_T_CELL_ACTIVATION ssGSEA enrichment score in TCGA,CGGA325, CGGA693 and GSE16011 GBM cohorts(B-E), also in TCGA, CGGA325, CGGA693 and GSE16011 All Grade cohorts(F-I) (Spearman correlation, All *P* < 0.001).

10.7717/peerj.12547/supp-2 Supplemental Information 2 Relationships between the risk score and clinical characteristics in validation cohorts. A-C: High risk score was distributed in higher grade gliomas. D-I: The higher risk score patients were mainly concentrated in the mesenchymal subtype(D-F) and IDH1 wild type(G-I) in GBM validation cohorts (Student’s t test, * means *P* < 0.05, ** means *P* < 0.01, *** means *P* < 0.001, **** means *P* < 0.0001)

10.7717/peerj.12547/supp-3 Supplemental Information 3 The prognostic value of the risk score in validation cohorts. Patients with high risk score in GBM(A-C), LGG(D-F) and All grades(G-I) had poor prognosis in validation cohorts (A: *P*-value = 0.02, B: *P*-value=0.063, C: *P*-value = 0.014, D-I: *P*-value <0.001, Log-rank test).

10.7717/peerj.12547/supp-4 Supplemental Information 4 The forest plots and calibration plots of validation cohorts. A-F: Forest plots of univariate and multivariate Cox regression analysis of the risk score in validation cohorts. G-H: Calibration plots of GBM validation sets.

10.7717/peerj.12547/supp-5 Supplemental Information 5 Genetic functional enrichment analysis of the differentially expressed genes between the high and low risk score groups in validation cohorts. A-B: Bubble plots of GO and KEGG pathway enrichment analyses’ results. C-F: Corplots showed that the risk score was positively correlated with immune-related Gene Programs enrichment scores in TCGA, CGGA325, CGGA693 and GSE16011 GBM cohorts. G-H: Validation cohorts’ GSEA analyses showed that immune-related gene sets were significantly enriched in the high risk score group.

10.7717/peerj.12547/supp-6 Supplemental Information 6 The risk score was negatively correlated with tumor Purity, and positively correlated with Immune Score and Stromal Score in validation cohorts. A-C: Correlations between the risk score and purity in validation cohorts. D-F: Correlations between the risk score and immune score in validation cohorts. G-I: Correlations between the risk score and stromal score in validation cohorts (Spearman correlation, All *P* < 0.001).

10.7717/peerj.12547/supp-7 Supplemental Information 7 The heatmap of metagenes results showed that high risk score was associated with increasing multiple immune cells in the tumor in validation cohorts. A: The metagenes result in CGGA325-seq GBM cohort. B: The metagenes result in CGGA693-seq GBM cohort. C: The metagenes result in GSE16011 GBM cohort.

10.7717/peerj.12547/supp-8 Supplemental Information 8 Correlations among the anti-tumor immunity, the pro-tumor suppression and the risk score. A-C: Metagenes results showed that there was a positive correlation between the anti-tumor immunity score and the pro-tumor suppression score, and both were higher in the high risk score group in validation cohorts. D-I: The risk score was significantly positively correlated with both the anti-tumor immunity score and the pro-tumor suppression score in validation cohorts (Spearman correlation, All *P* < 0.001).

10.7717/peerj.12547/supp-9 Supplemental Information 9 MCP counter compared differences in various types of immune cells between the high and low risk score groups in validation cohorts. A: The MCP counter result in CGGA325-seq GBM cohort. B: The MCP counter result in CGGA693-seq GBM cohort. C: The MCP counter result in GSE16011 GBM cohort (Student’s t test).

10.7717/peerj.12547/supp-10 Supplemental Information 10 In validation cohorts, relationships between the risk score and immune checkpoints expression, and the sensitivity of patients to immunotherapy. A: Proportion differences of Tumor Microenvironment Immune Types between the high and low risk score groups’ patients in validation cohorts. B-D: The risk score showed a positive correlation with the expression level of immune checkpoints: PD-1, PD-L1, CTLA-4, TIM-3, IDO-1 and LAG-3 in validation cohort.

10.7717/peerj.12547/supp-11 Supplemental Information 11 Combined the T cell activation priming score and the risk score to predict the sensitivity of patients with GBM to immunotherapy in validation cohorts. A-C: In validation cohorts the priming score showed a positive correlation with the risk score as well, and TIDE results also showed that patients were sensitive to immunotherapy while the priming scores were high and the risk scores were low (Spearman correlation, All *P* < 0.001). D-F: There was a significant difference in TIDE results between the high-risk/low-priming group and the high-priming/low-risk group in validation cohorts (D: *P*-value <0.0001, chi-square test; E: *P*-value <0.0001, chi-square test; F: *P*-value = 0.8845, chi-square test). G-I: The immunotherapy score was significantly associated with TIDE results in validation cohorts (G: *P*-value <0.0001, chi-square test; H: *P*-value <0.0001, chi-square test; I: *P*-value =0.0019, chi-square test).

10.7717/peerj.12547/supp-12 Supplemental Information 12 Gene Ontology terms of T-cell activation and T-cell activation priming, univariate Cox regression results of tumor-related T-cell activation genes, and the list of abbreviations.

10.7717/peerj.12547/supp-13 Supplemental Information 13 Results of GO analyses, KEGG pathways analyses and gene sets enrichment analyses.

10.7717/peerj.12547/supp-14 Supplemental Information 14 Patients’ scores of the five cohorts.

## Additional Information and Declarations

### Data Availability

The following information was supplied regarding data availability: The Glioblastoma (GBM) mRNA-seq dataset and TCGA Glioblastoma (GBM) phenotypes dataset were retrieved from TCGA (https://tcga-data.nci.nih.gov). The microarray is available at GSE16011. The CGGA325 RNA-seq cohort (Part C, DataSet ID: mRNAseq_325) and CGGA693 RNA-seq cohort (Part B, DataSet ID: mRNAseq_693) are available at the Chinese Glioma Genome Atlas: http://www.cgga.org.cn/download/20191128/download.jsp. The patients’ scores of the five cohorts involved in the article are available in the Supplementary File.
