# Supplementary figures and images for: Development of a T-cell activation-related module with predictive value for the prognosis and immune checkpoint blockade therapy response in glioblastoma
Source: PeerJ. 2021 Dec 22;9:e12547. doi: 10.7717/peerj.12547 (PMC8710057; doi:10.7717/peerj.12547)

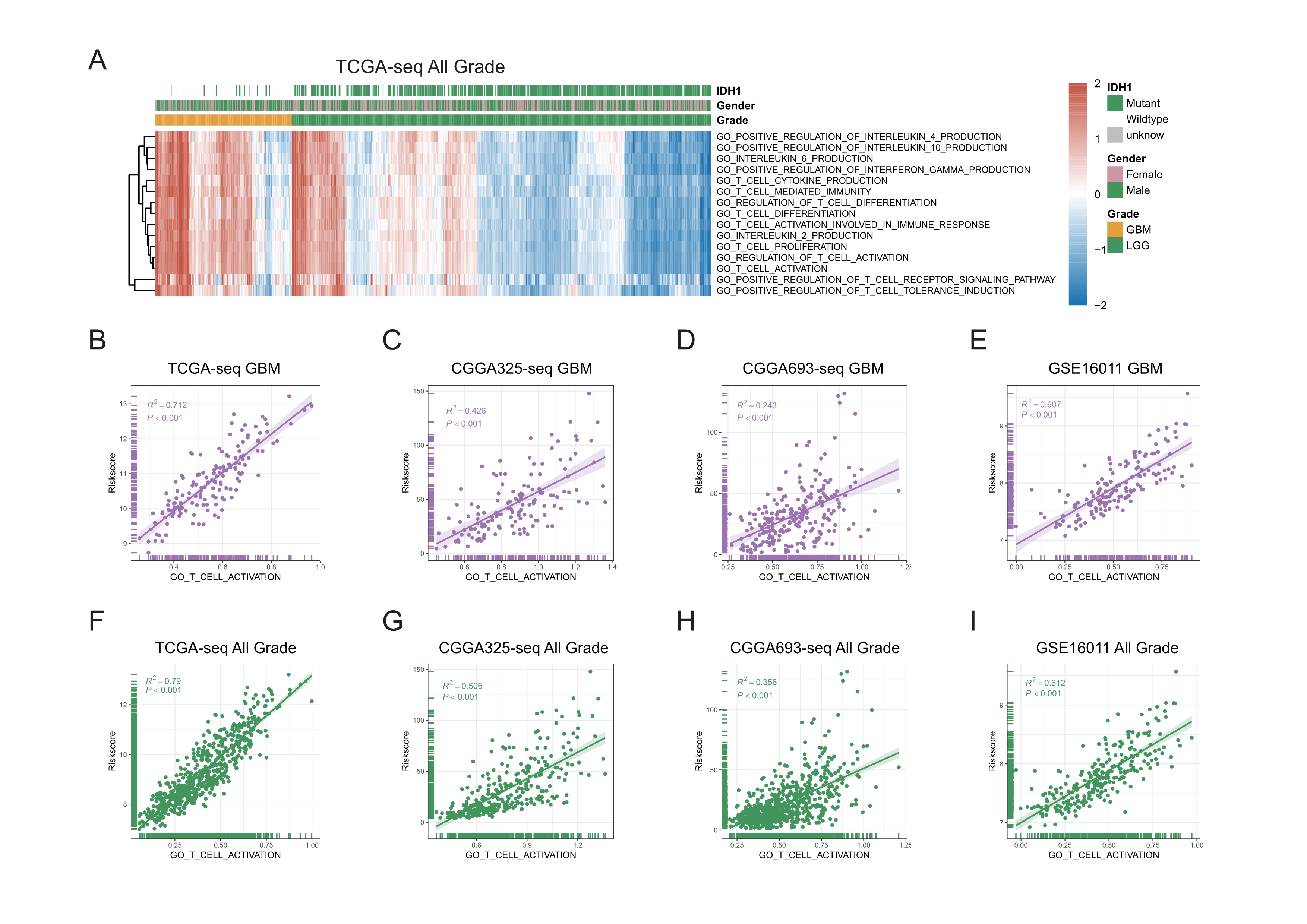

Supplement: Supplemental Information 1 — A: GO_T_CELL_ACTIVATION and its child terms’ ssGSEA enrichment scores distributed differently in TCGA glioma patients, and were significantly higher in GBM patients. B-I: T cell activation related risk score was significantly positively correlated with GO_T_CELL_ACTIVATION ssGSEA enrichment score in TCGA,CGGA325, CGGA693 and GSE16011 GBM cohorts(B-E), also in TCGA, CGGA325, CGGA693 and GSE16011 All Grade cohorts(F-I) (Spearman correlation, All P < 0.001). [file peerj-09-12547-s001.jpg]

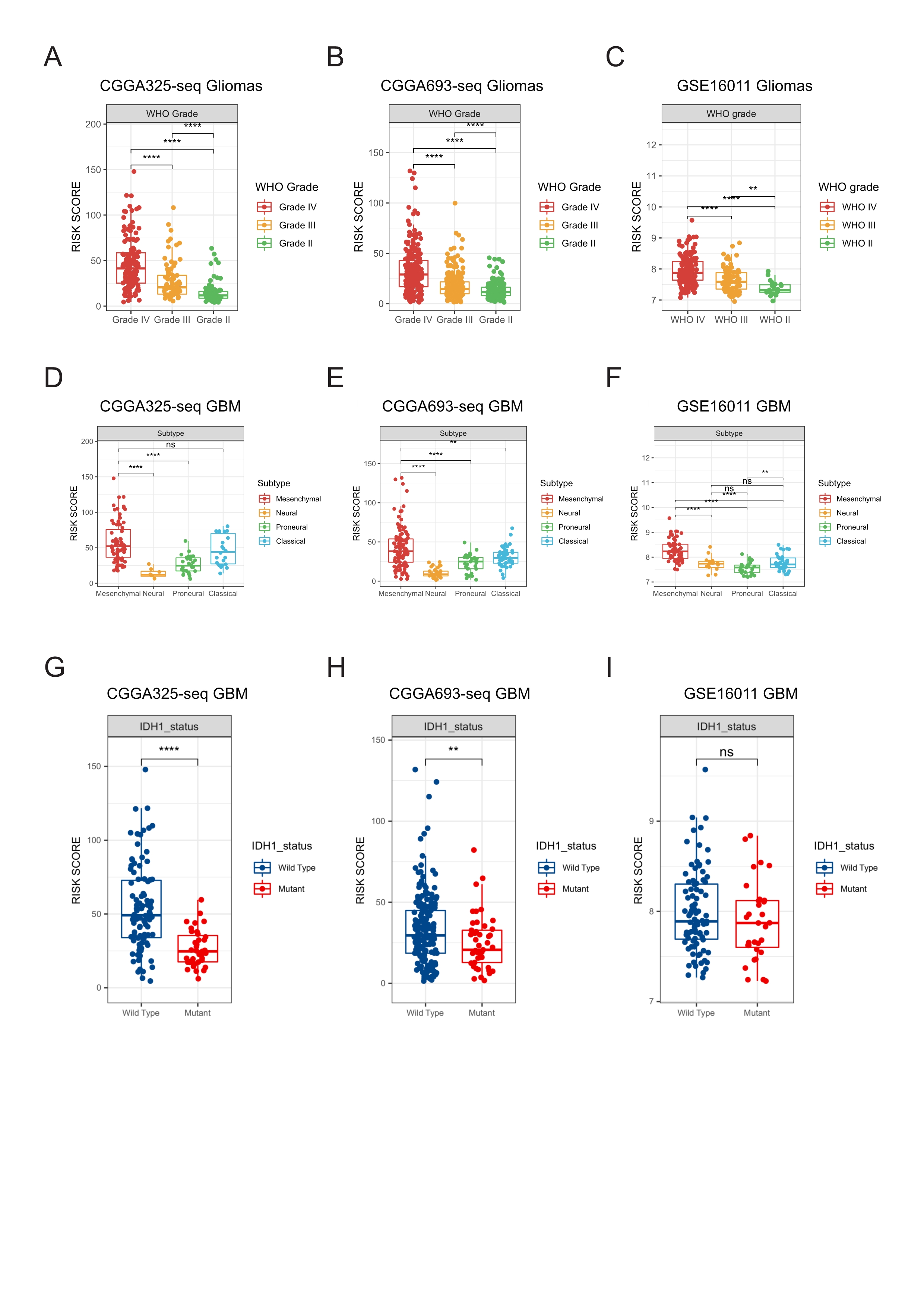

Supplement: Supplemental Information 2 — A-C: High risk score was distributed in higher grade gliomas. D-I: The higher risk score patients were mainly concentrated in the mesenchymal subtype(D-F) and IDH1 wild type(G-I) in GBM validation cohorts (Student’s t test, * means P < 0.05, ** means P < 0.01, *** means P < 0.001, **** means P < 0.0001) [file peerj-09-12547-s002.jpg]

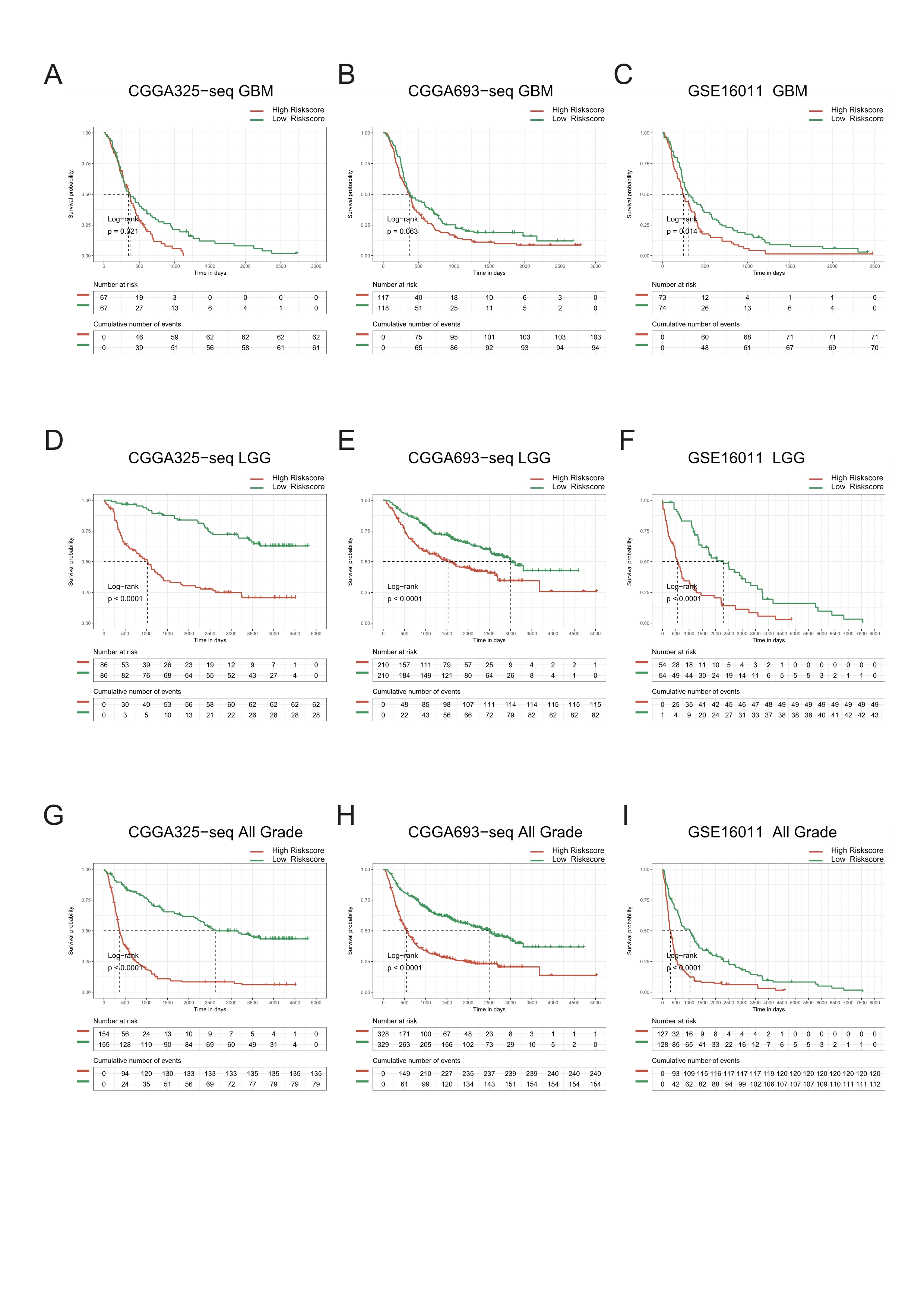

Supplement: Supplemental Information 3 — Patients with high risk score in GBM(A-C), LGG(D-F) and All grades(G-I) had poor prognosis in validation cohorts (A: P-value = 0.02, B: P-value=0.063, C: P-value = 0.014, D-I: P-value <0.001, Log-rank test). [file peerj-09-12547-s003.jpg]

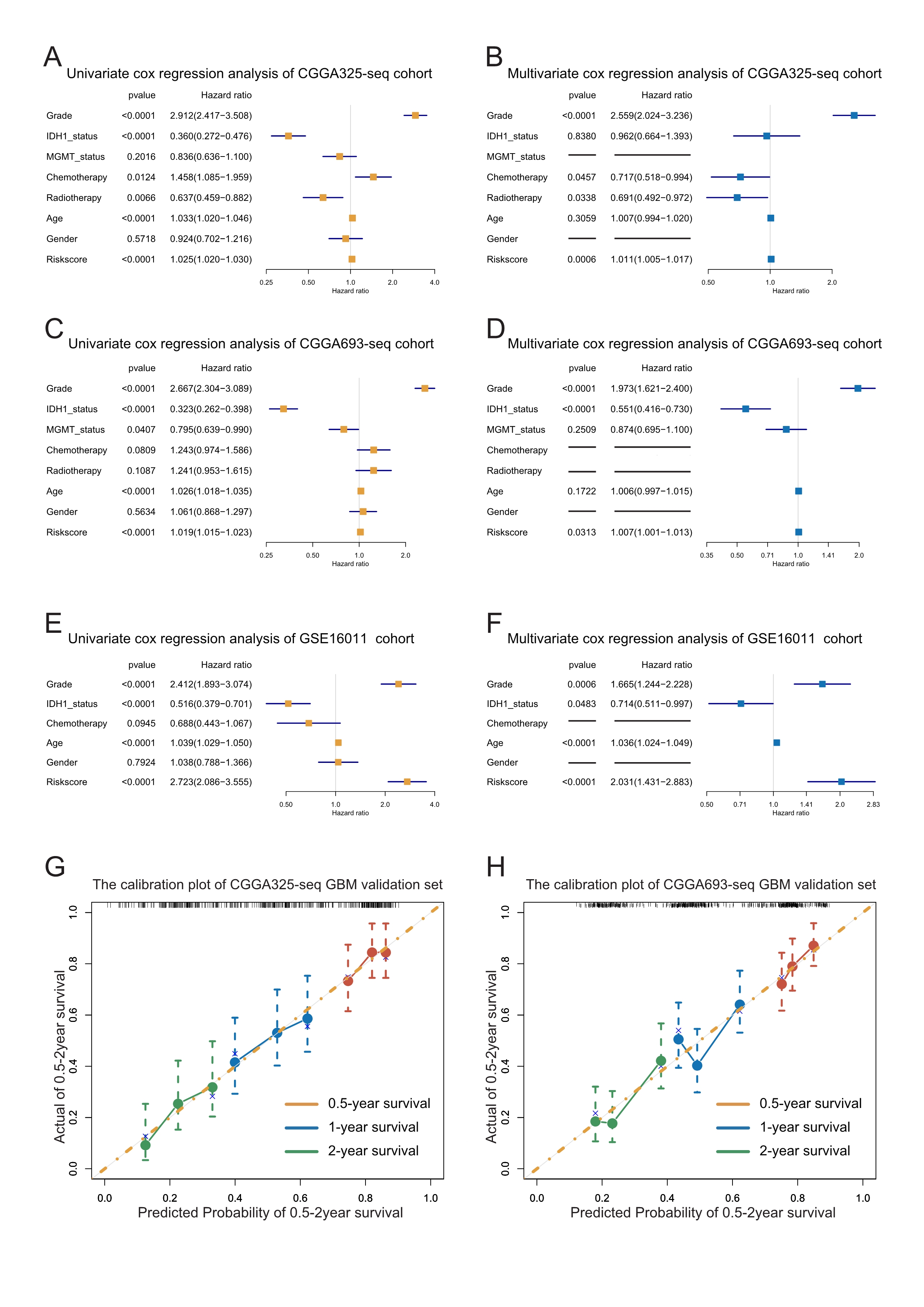

Supplement: Supplemental Information 4 — A-F: Forest plots of univariate and multivariate Cox regression analysis of the risk score in validation cohorts. G-H: Calibration plots of GBM validation sets. [file peerj-09-12547-s004.jpg]

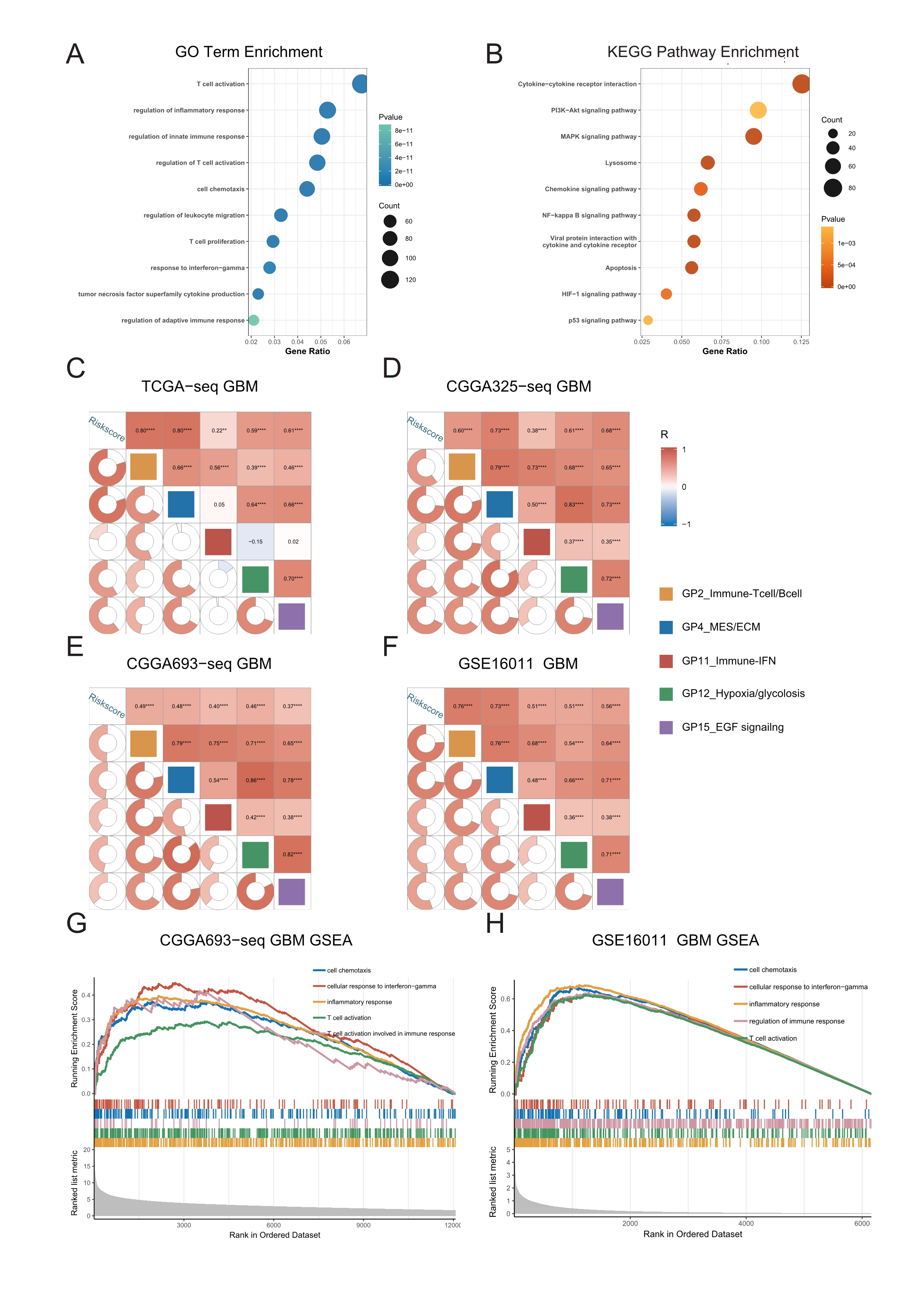

Supplement: Supplemental Information 5 — A-B: Bubble plots of GO and KEGG pathway enrichment analyses’ results. C-F: Corplots showed that the risk score was positively correlated with immune-related Gene Programs enrichment scores in TCGA, CGGA325, CGGA693 and GSE16011 GBM cohorts. G-H: Validation cohorts’ GSEA analyses showed that immune-related gene sets were significantly enriched in the high risk score group. [file peerj-09-12547-s005.jpg]

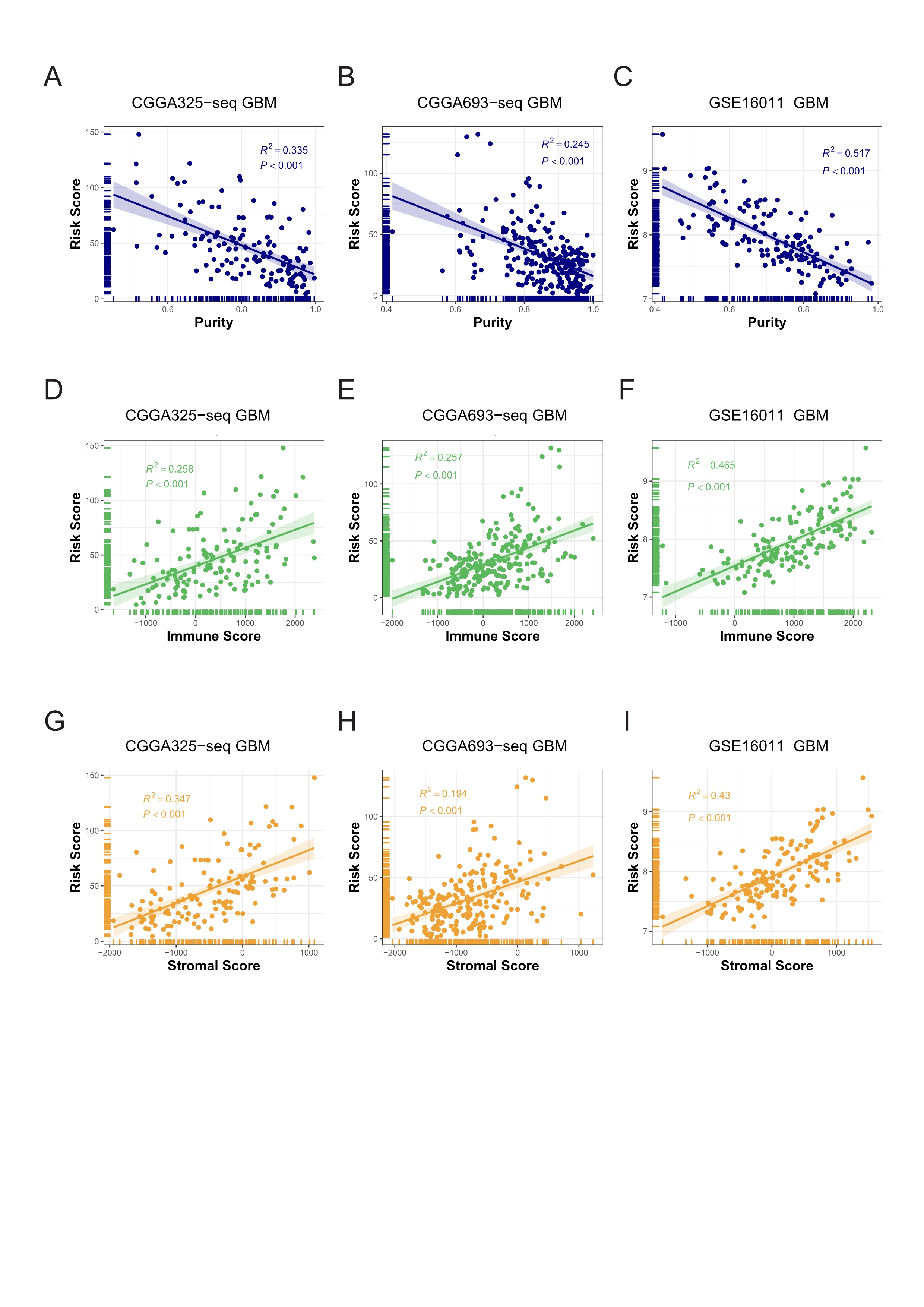

Supplement: Supplemental Information 6 — A-C: Correlations between the risk score and purity in validation cohorts. D-F: Correlations between the risk score and immune score in validation cohorts. G-I: Correlations between the risk score and stromal score in validation cohorts (Spearman correlation, All P < 0.001). [file peerj-09-12547-s006.jpg]

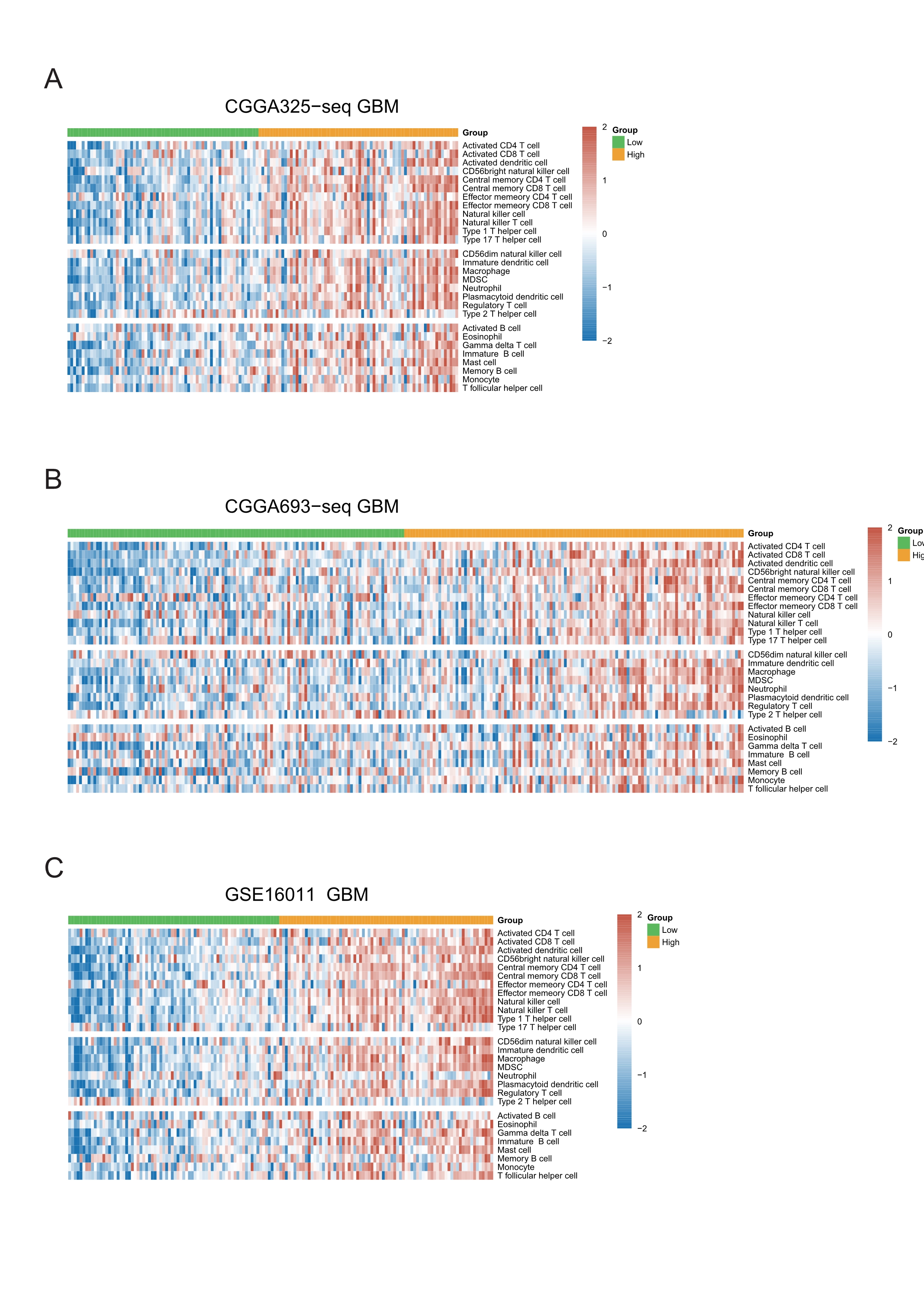

Supplement: Supplemental Information 7 — A: The metagenes result in CGGA325-seq GBM cohort. B: The metagenes result in CGGA693-seq GBM cohort. C: The metagenes result in GSE16011 GBM cohort. [file peerj-09-12547-s007.jpg]

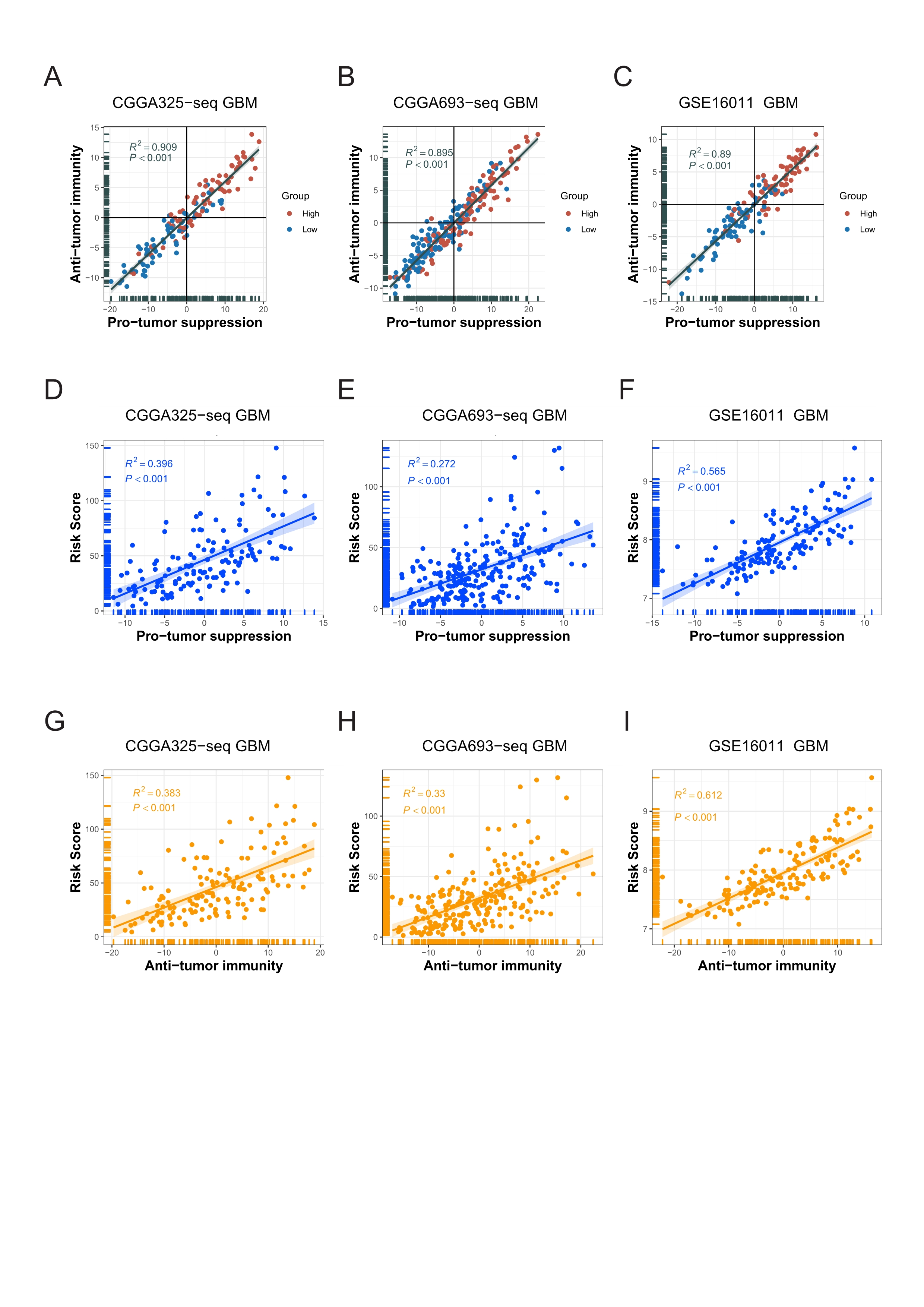

Supplement: Supplemental Information 8 — A-C: Metagenes results showed that there was a positive correlation between the anti-tumor immunity score and the pro-tumor suppression score, and both were higher in the high risk score group in validation cohorts. D-I: The risk score was significantly positively correlated with both the anti-tumor immunity score and the pro-tumor suppression score in validation cohorts (Spearman correlation, All P < 0.001). [file peerj-09-12547-s008.jpg]

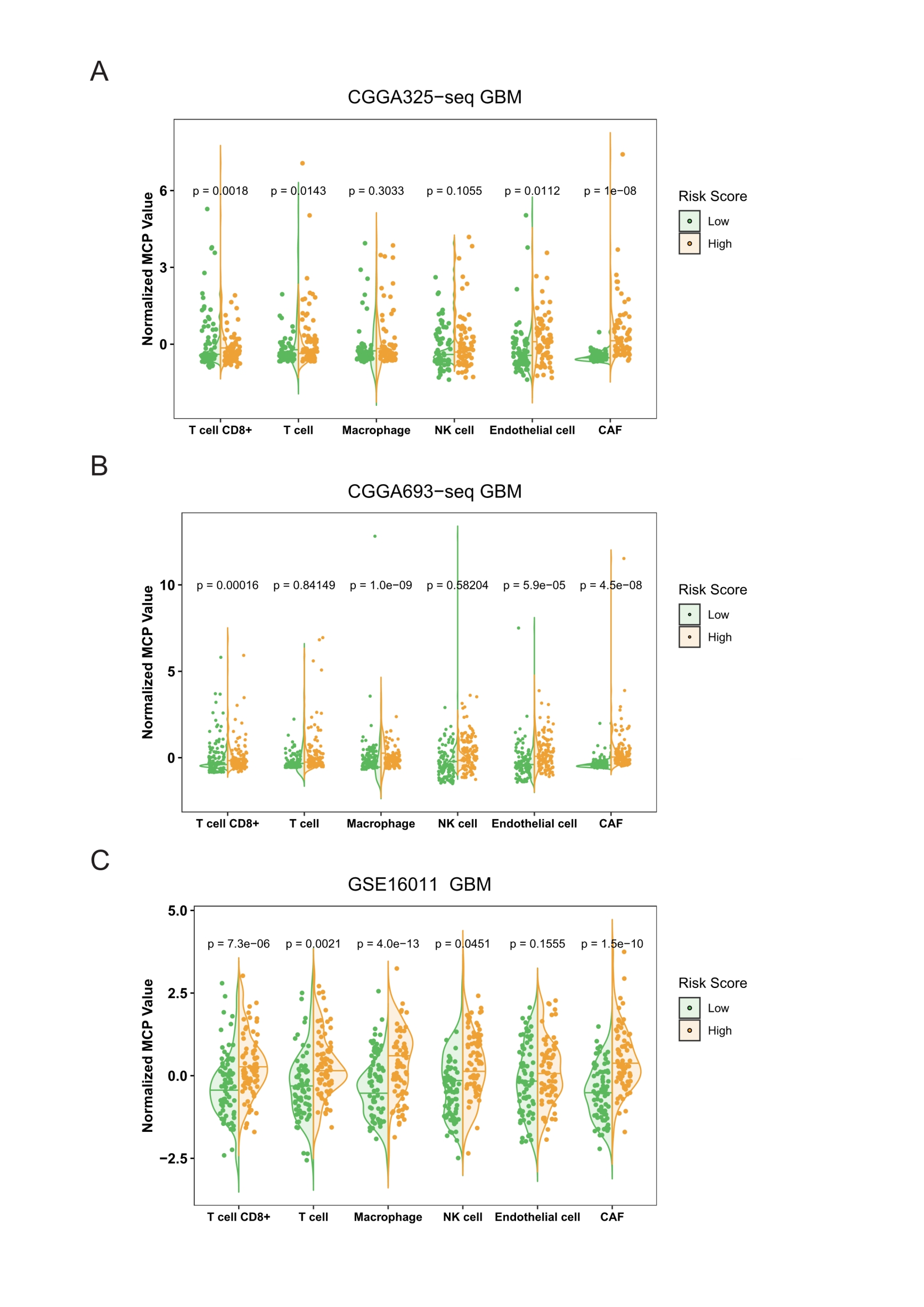

Supplement: Supplemental Information 9 — A: The MCP counter result in CGGA325-seq GBM cohort. B: The MCP counter result in CGGA693-seq GBM cohort. C: The MCP counter result in GSE16011 GBM cohort (Student’s t test). [file peerj-09-12547-s009.jpg]

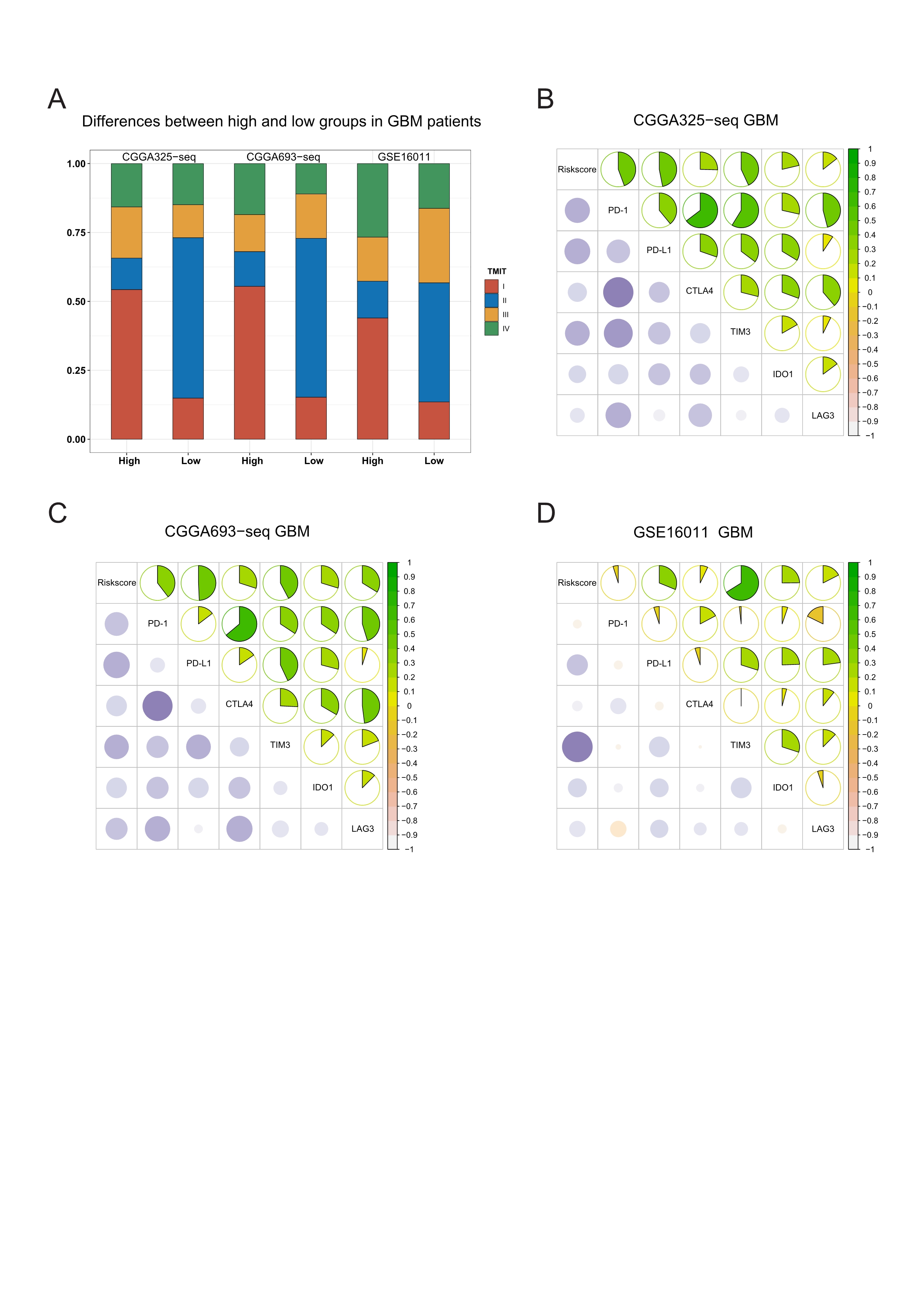

Supplement: Supplemental Information 10 — A: Proportion differences of Tumor Microenvironment Immune Types between the high and low risk score groups’ patients in validation cohorts. B-D: The risk score showed a positive correlation with the expression level of immune checkpoints: PD-1, PD-L1, CTLA-4, TIM-3, IDO-1 and LAG-3 in validation cohort. [file peerj-09-12547-s010.jpg]

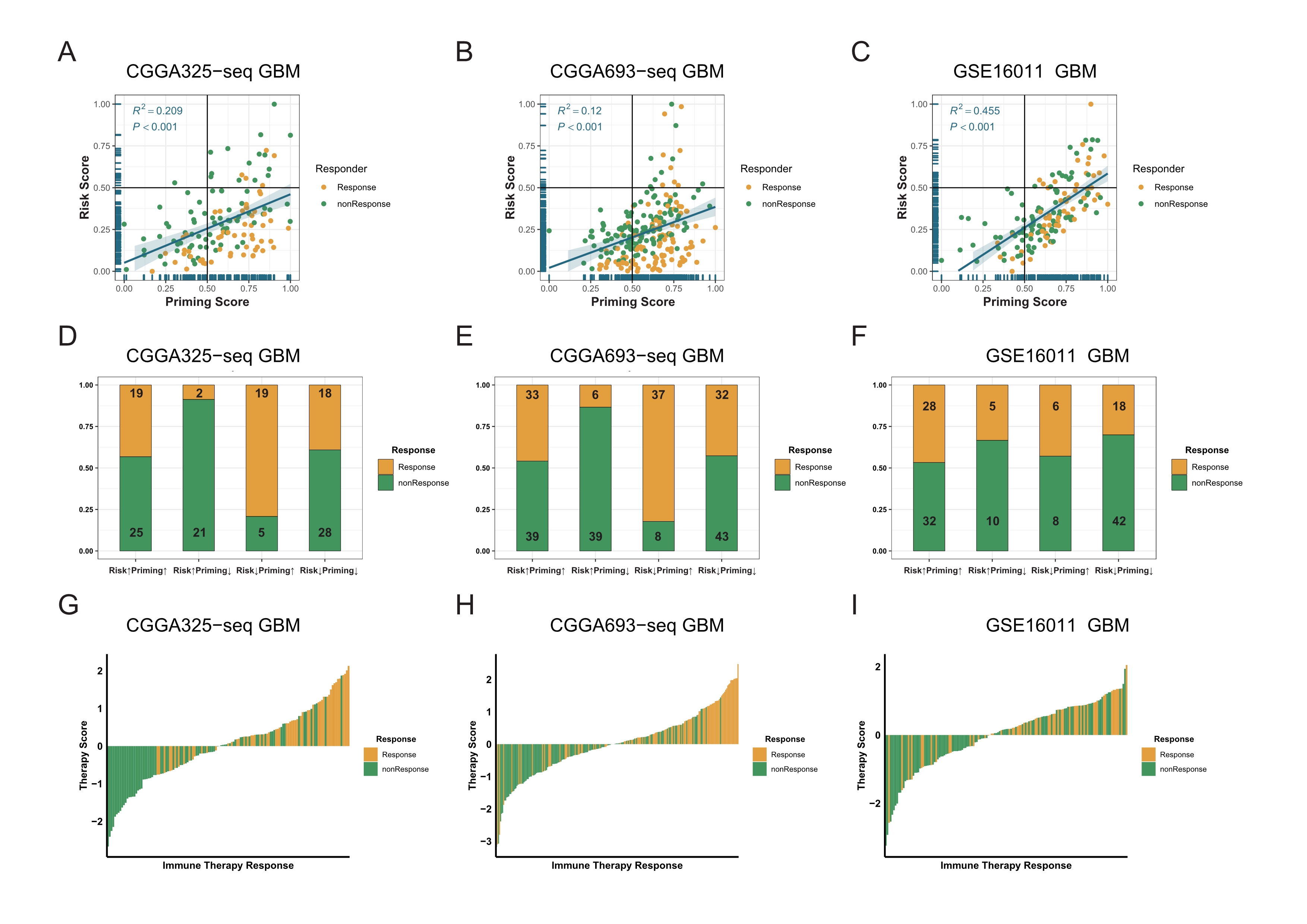

Supplement: Supplemental Information 11 — A-C: In validation cohorts the priming score showed a positive correlation with the risk score as well, and TIDE results also showed that patients were sensitive to immunotherapy while the priming scores were high and the risk scores were low (Spearman correlation, All P < 0.001). D-F: There was a significant difference in TIDE results between the high-risk/low-priming group and the high-priming/low-risk group in validation cohorts (D: P-value <0.0001, chi-square test; E: P-value <0.0001, chi-square test; F: P-value = 0.8845, chi-square test). G-I: The immunotherapy score was significantly associated with TIDE results in validation cohorts (G: P-value <0.0001, chi-square test; H: P-value <0.0001, chi-square test; I: P-value =0.0019, chi-square test). [file peerj-09-12547-s011.jpg]
